# Overlooked Short Toxin-Like Proteins: A Shortcut to Drug Design

**DOI:** 10.3390/toxins9110350

**Published:** 2017-10-29

**Authors:** Michal Linial, Nadav Rappoport, Dan Ofer

**Affiliations:** 1 Department of Biological Chemistry, Silberman Institute of Life Sciences, The Hebrew University of Jerusalem, Jerusalem 91904, Israel; ddofer@gmail.com; 2 Institute for Computational Health Sciences, UCSF, San Francisco, CA 94158, USA; nadav.rappoport@ucsf.edu

**Keywords:** neurotoxin, protein families, disulfide bonds, antimicrobial peptide, ion channel inhibitor, ClanTox, complete proteome, comparative proteomics, machine learning, insects

## Abstract

Short stable peptides have huge potential for novel therapies and biosimilars. Cysteine-rich short proteins are characterized by multiple disulfide bridges in a compact structure. Many of these metazoan proteins are processed, folded, and secreted as soluble stable folds. These properties are shared by both marine and terrestrial animal toxins. These stable short proteins are promising sources for new drug development. We developed ClanTox (classifier of animal toxins) to identify toxin-like proteins (TOLIPs) using machine learning models trained on a large-scale proteomic database. Insects proteomes provide a rich source for protein innovations. Therefore, we seek overlooked toxin-like proteins from insects (coined iTOLIPs). Out of 4180 short (<75 amino acids) secreted proteins, 379 were predicted as iTOLIPs with high confidence, with as many as 30% of the genes marked as uncharacterized. Based on bioinformatics, structure modeling, and data-mining methods, we found that the most significant group of predicted iTOLIPs carry antimicrobial activity. Among the top predicted sequences were 120 termicin genes from termites with antifungal properties. Structural variations of insect antimicrobial peptides illustrate the similarity to a short version of the defensin fold with antifungal specificity. We also identified 9 proteins that strongly resemble ion channel inhibitors from scorpion and conus toxins. Furthermore, we assigned functional fold to numerous uncharacterized iTOLIPs. We conclude that a systematic approach for finding iTOLIPs provides a rich source of peptides for drug design and innovative therapeutic discoveries.

## 1. Introduction

Short proteins are strong candidates for peptide-based therapy and drug development [1,2,3]. The search for peptide-based drugs is driven by the urge to improve specificity and affinity over classical drugs [4]. At present, the search for new leads for peptide therapy is mostly restricted to known peptides that act as hormones, neuropeptides, and growth factors [5,6,7].

Venomous proteins are found in diverse taxonomical branches including scorpions, snakes, spiders, and marine cone snails [8]. Venomous animals have developed sophisticated array of delivery systems for defense and offense. Evolutionary studies suggest that venomous toxins often reuse common folds that are abundant in the animal phyla (e.g., lipases [9]). Sequences of short proteins that are characterized by having numerous cysteines often fold into compact, stable structural folds. The resulting different folds are often found in proteins that carry diverse functions (e.g., lectins, protease, and protease inhibitors [10]). Venomous organisms are sporadically scattered within the phylogenetic tree of life. Venomous proteins represent cases of both divergent and convergent evolution, as well as repeated use of several existing, successful and abundant folds. However, the pool of bioactive short peptides resembling animal toxins is larger than anticipated [11]. The toxins’ innovation is exemplified by their high degree of sequence variation and broad specificities, with only minimal alterations in the structural scaffolds [12].

In recent years, additional bioactive peptides were identified via systematic searches in the transcriptomes and proteomes of venomous animals [13,14]. Secreted short proteins from venomous glands may include hundreds of poorly studied bioactive peptides [6]. Approximately 2000 toxins out of an estimated >70,000 bioactive peptides have been identified in the genus Conus to date [15]. Evolutionary perspective based on the huge sequence diversity among toxins provides a rich source for rational protein design [16,17].

Toxins are extremely varied in their functions and mode of action. The potency of toxins’ function is associated with an extremely broad collection of ion channel inhibitors (ICIs), phospholipases, protease inhibitors, disintegrins, membrane pore inducers, and more [18]. Some animal toxins affect the most basic cellular properties [19]. Examples include the non-reversible effect of amphipathic peptides on the membrane integrity [20] from spider venom [21] to marine hydrozoan toxins [22]. These toxins may cause non-specific hemolysis [23]. However, most toxin proteins act via highly specific binding to their cognate molecular target, making them attractive for drug design. The neuronal [24] and immune systems [25] are often affected by toxin-target molecular recognition. A well-studied example for reuse of a fold that acts on numerous receptors of the cholinergic system was described by Gibbons et al. [26]. The three-finger proteins (TFP) fold is found in numerous mammalian proteins acting in the innate immune system [27], and was also identified as Elapidae α-neurotoxins [28,29]. Two striking examples of human toxin-like proteins are Lynx1 [30] and SLURP-1 [31]. These are human proteins that possess similarity to snake α-neurotoxins, and modulate nicotinic acetylcholine receptors (nAChR), as does the snake α-neurotoxins. The identification of SLURP-1 as a neuromodulator has contributed to the understanding of the genetic effect of the Mal de Meleda disease, a skin disease that results from over activation of TNF-alpha [31].

Many short bioactive molecules are ion channels blockers (ICIs) and toxins with antimicrobial activity [32]. ICIs constitute the most widely studied group of toxins. A large group of ICIs whose evolution has been studied are the K^+^ ICIs [33]. It is estimated that more than 10 different structural folds and 40 structural families represent this extremely diverse (structurally and evolutionally) group [34]. In spite of that, two amino residues are critical for all K^+^ ICIs’ function: Lys and a Tyr/Phe, known as the functional dyad [35]. Surprisingly, even though these residues appear in very different positions along the sequences of K^+^ ICIs, the solved structures show they are similarly aligned in space relatively to each other [36]. The same principle of sequence plasticity and structural rigidity apply for ICIs that affect other channels (e.g., [37,38,39,40]). Different ICIs targeting the same channel can vary in both sequence and structural folds [41].

The evolutionary mechanisms underlying the extreme diversity of toxins have been investigated [42]. Direct approaches for assessing the rapid mutation rate of a variety of toxins sharing the same fold have been reported (e.g., for phospholipases A2 [43]). TFP topology is also a strong example of the accelerated evolution and functional diversification reported for many snake toxins [44]. 3D complexes of short toxins and their cognate channels provide the best lead for the design of toxin-based pharmaceutical agents (e.g., [45]). A number of short toxins are already being used in the clinic for pain management [46], antiviral and antibacterial applications [47].

A common ICI design principle is conserved spacing, and the number of cysteines that form a stable scaffold in a few disulfide bridges [11]. In many cases, the core elements of the fold remain untouched by the preservation of at least two cysteine bridges, while the surfaces of the toxins undergo a natural dynamic adaptive evolution process. The extreme stability of the cysteine knot motif in peptide toxins makes these folds attractive for molecular engineering and drug design [48].

Based on the observation that many short animal toxins are rich in cysteines [49,50], we focused on a subset of short proteins (<75 amino acids) that can be used for discoveries towards peptide therapy [51]. The goal of our study is to present a systematic approach for identifying insects’ toxin-like proteins TOLIPs (iTOLIPs). We analyzed a large number of published proteomes [52]. A rich catalogue of short bioactive proteins will have the potential to benefit the pharma and medical communities that seek new leads for drugs [53].

Insects represent one of the most diversified metazoan phyla. Many insect species evolved in unique ecological niches (e.g., parasitoid wasp) [54], and exhibit complex social behavior with rapidly evolving genomes [55,56]. In this study, we show that despite limited sequence similarity between short sequences, many toxin-like candidate sequences have been revealed via a machine learning predictor (ClanTox [57]). ClanTox was trained only on features extracted from ion channels inhibitors (ICI) from venomous proteins, for identifying TOLIPs. Using a rigorous bioinformatics and structural modeling scheme, we assigned a potential functional relevance for numerous iTOLIPs. We present dozens of new candidates for peptide-based therapy and discuss their potential for drug design.

## 2. Results and Discussion

### 2.1. Thousands of Toxin-Like Secreted Short Proteins in Insects 

UniProtKB is the largest existing proteomic database (about 90 million sequences, August 2017) and is the main source of new templates for drug development. In recent years many new genomes have been sequenced including >30 insects. Despite a tsunami of genome sequences, only a few model organisms (e.g., *Drosophila melanogaster*) have high quality, manually annotated proteomes. While DNA sequencing quality has improved dramatically, current gene finding methodologies are still geared towards finding transcripts based on length (usually >100 amino acids, AA). Functional inference of genes’ function from a transcribed genome remains an unsolved challenge [58]. Short proteins often have missing or faulty annotations (e.g., [59]).

**Figure 1:**
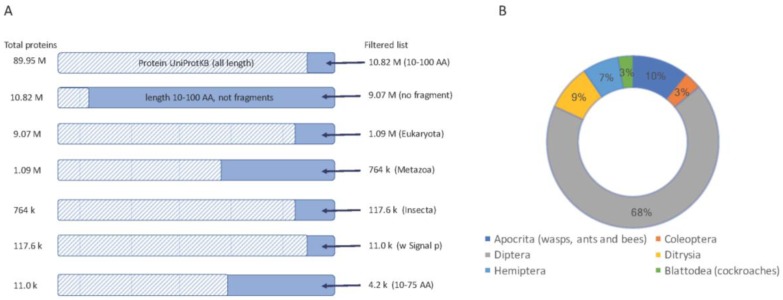
Selection of short secreted proteins from insect proteomes. (**A**) A sequence of filtration steps for protein sequences from UniProtKB is shown (top to bottom). Each step shows the number of proteins (left) and the resulting protein (right). The dashed bar marks the fraction of the data that is excluded from the following step. Sequences marked as “fragments” by UniProtKB were excluded. The final set used in this study includes proteins from Insecta with a “signal peptide” sequence annotation keyword, a restricted length of 10–100 AA and a further selection for proteins length of 10–75 AA. (**B**) A partition of the main orders of insects and their representation from the set of about 11,000 proteins.

We focused our discovery platform on short proteins. For the rest of the analyses we considered two thresholds on the proteins’ length: (i) proteins of length <100 AA (Figure 1); (ii) a subset of shorter proteins, length <75 AA, that are attractive for drug development.

We started with all proteins shorter than 100 AA (after removing all fragmented proteins), restricted to the insects’ taxon, which resulted in ~117,600 proteins. Of these, 11,000 proteins were predicted to be secreted, and thus function in the extracellular space (Figure 1A).

Analyzing the ~11,000 protein’-origins show that the proteomes of major orders of insects are biased towards the previously sequenced genomes (Figure 1B). Diptera, which includes mosquitos and flies, dominates the collection (68%). The rest of the candidate short proteins belong to Hymenoptera (mostly bees, wasp, and ants, 10%), Ditrysia (including moth, bumblebee, and butterfly, 9%) and a smaller amount of Hemiptera (e.g., aphids), Coleoptera (mostly beetles) and Blattodea (mostly termites).

While most insects are not venomous [19], some bees, ants, and wasps developed mechanisms to release their venomous proteins and toxic peptides. Many of the short proteins are uncharacterized (see discussion in [56]). Moreover, annotations of genes from fast evolving organisms are often missing. Due to these fast evolutionary innovation in many insects, we anticipate a rich repertoire of overlooked bioactive peptides [60] and iTOLIPs [61].

We used ClanTox [57] to investigate the abundance of iTOLIPs among the 11,000 short, secreted proteins (<100 AA). To this end, we divided the protein according to the major orders of insects, and further investigated the ClanTox predictions, according to the confidence level of the predictor (marked as P1–P3, see Methods). We have previously shown that many valid TOLIPs are identified at all confidence levels, including the least confident one (P1, see Methods, [57]). ClanTox was trained only on ICIs from venomous animals for seeking TOLIPs from all organisms. While it was trained on a limited function, predictions are associated with a much broader spectrum of functions that specify known toxins and proteins with no known homologues in venoms [11].

**Figure 2:**
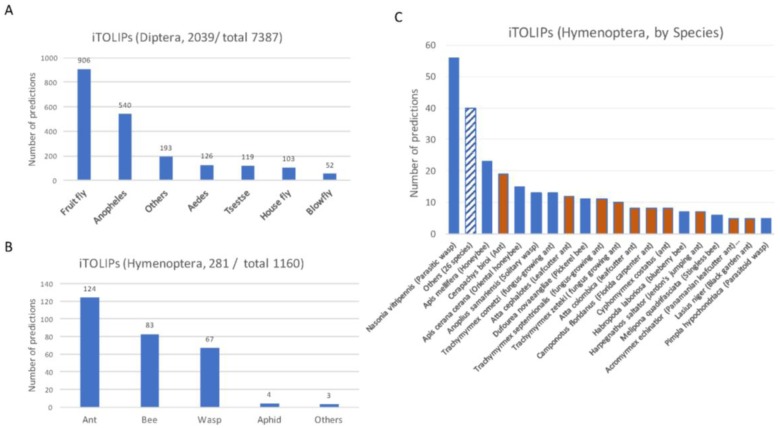
**Partition of ClanTox prediction for mini-proteins of toxin-like proteins from insects (iTOLIPs)**. The fraction of iTOLIPs that was identified as iTOLIPs by ClanTox is shown for the orders Diptera (**A**), and Hymenoptera (**B**). Only major genus representatives are shown. The total numbers indicate the number of sequences that were introduced to ClanTox. (**C**) A detailed partition of the species that are associated with iTOLIPs. Only species having ≥5 proteins are listed. The dashed bar is an aggregation of iTOLIPs from 26 different species. Orange bar are different ant species, and blue bars are other representatives of Hymenoptera.

Figure 2 shows the results from ClanTox prediction with iTOLIPs cover the two largest orders of insects, the Diptera (Figure 2A) and Hymenoptera (Figure 2B). A bias in the prediction towards model organisms is evident. The iTOLIPs from Drosophilae (fruit fly) accounts for 44% of the predicted sequences. Still, >1000 sequences are detected in less studied organisms, such as the Tsetse fly, Aedes, blowfly, and more (Figure 2A). The fraction of iTOLIPs among the cysteine rich short proteins from Hymenoptera (wasp, bees, and ants) is 24%. The high number of iTOLIPs from ant proteomes is a reflection of the many recently sequenced ant genomes (Figure 2C) [56]. Note that the number of predictions from *Nasonia vitripennis* (Parasitic wasp) is disproportionally high. Of 145 *Nasonia vitripennis’*-short proteins, 57 (39%) were predicted as iTOLIPs (Figure 2C).

From a therapeutic perspective, often, the shorter the protein, the easier it is to produce it synthetically, and to introduce it to laboratory and clinical trials. We restricted the search to 4181 sequences are shorter than 75 AA (Figure 1A).

Figure S1 shows the distribution of the 4181 sequences according to ClanTox’s prediction confidence (N, P1–P3, see Methods). Note that most proteins (76%) are predicted as negative, and do not comply with the definition of iTOLIPs (Clantox’s label N stands for—“not a toxin-like”). The high confidence predictions (P3, top prediction for Toxin-like) include 379 proteins (9%, Figure S1). The rest of the analyses will focus on these high confidence-predicted iTOLIPs (P3).

Table 1 shows the partition of the top predicted iTOLIPs among the major orders of insects. The most outstanding observation is the abundance of iTOLIPs in termites (52%), and the low discovery of top prediction iTOLIPs among Ditrysia (5%). A list of 379 predicted sequences is available (Table S1).

### 2.2. Most iTOLIP Mini-Proteins Resemble Antibacterial and Antifungal Peptides

Antimicrobial peptides (AMPs) are very abundant among insects [62]. At present, >150 insect AMPs have been identified [63]. A total of 121 peptides out of 379 iTOLIPs are from the Blattodea order, and named by UniProtKB as “termicin”. Among the top predicted iTOLIPs, these proteins comprise the largest group. Termicins are restricted to the order Blattodea (termites and cockroaches). These are a collection of secreted AMP mini-proteins (25–40 AA), sharing a moderate sequence similarity. A termicin-like peptide (25 AA) from the cockroach *Eupolyphaga sinensis* exhibits anti-fungal activity, and a weak activity against bacteria [63]. We hypothesize that other sequences among the al iTOLIPs resemble antimicrobial proteins and potentially act as such.

**Figure 3:**
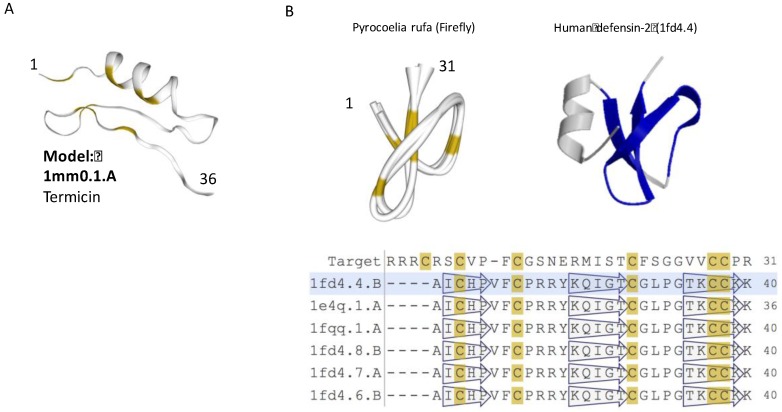
**Structural model of iTOLIPs with antifungal activity**. (**A**) The tertiary structure of D2D008_9NEOP from *Macrotermes barneyi* is shown. The structure is a representative of 120 related sequences of 35–36 AA identified as iTOLIPs. The model shows the α-helix stabilized next to two-stranded antiparallel β-sheet (called CSαβ). (**B**) A structural model for the mature Q95UJ8 protein (25–55 AA) from firefly (*Pyrocoelia rufa*) is shown. The best model for this sequence is the human defensin-2 protein (PDB:1fd4.4) (right). The light green shades indicate the overlap between the two proteins. Representatives for the structural model and their multiple sequence alignments are shown. The positions of the β-sheets are shown by the hollow arrows. Yellow color marks the position of the cysteines.

Structurally, termicin is characterized by three disulfide bridges forming a rigid fold. The tertiary structure of termicin contains an α-helical segment and a two-stranded antiparallel β-sheet (called cysteine-stabilized α-helix/β-sheet, CSαβ, Figure 3A). The structural motif of CSαβ is similar to that of short insect defensins. The cysteine positions and pairing suggest that despite a minimal sequence similarity with insect defensins, the structure is shared by all defensins [64]. Expending the analysis of ClanTox top predictions suggests that the AMP and defensin-like fold could be subjected for a design approach aiming to improve the peptide specificity in the current post-antibiotic era (Figure 3A).

The insect defensin protein is a shorter version of the human defensin-2 (Figure 3B). Furthermore, the human defensin’s N-terminal helix is completely missing in the firefly protein. It is plausible that functionality as an AMP comes from the core folded structure of (31 AA) of the firefly version of the defensin, and therefore, the N’-terminal helix is redundant (Figure 3B, light green shade). Structural variations of insect antimicrobial peptides illustrate the resemblance to a short version of the defensin fold. The diversity of AMP peptides in view of scorpion toxins had been extensively studied [65,66]. Defensins were also found among sponge, platypus, and scorpion toxins [67]. The assumption is that short specific structural motifs are used as templates by animal toxins [68]. Note that many additional versions of insect defensin genes are longer than 75 AA, and thus will not be further discussed [69,70].

**Figure 4:**
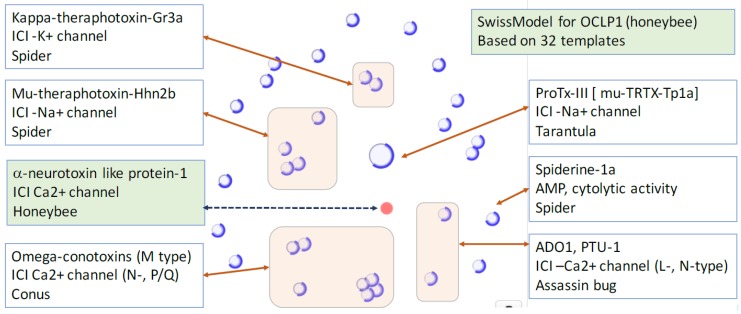
**Omega conotoxin –like protein 1 (OCLP1) and its similarity to structurally solved proteins used as templates**. The protein OCTP1 (red circle) is shown in view of a sequence similarity from the best SwissModel for H9KQJ7 (AA 26–74) from *Apis mellifera* (Honeybee). Each blue circle is one of the 32 template proteins. The functions of the listed proteins and the relevant organism are listed. ICI, ion channel inhibitor.

The other major shared function among the top predicted iTOLIPs (Table S1) is the antifungal activity associated with the many Drosomicin genes, including two large sets of DRO and DRS genes [71]. Drosomycins (DRS) are inducible antifungal peptides, and were isolated from the hemolymph of immune-challenged Drosophilae. A similar antifungal specificity applies for DRO1–DRO6 cassette, which responds to injury and microbial infection [72]. The DRS scaffold is a typical cysteine-stabilized α-helical and β-sheet (CSαβ) that specifies many of the known defensins (Figure 4). The hallmark of DRS gene is its extra-stability, which is gained by clamping the N’- and C’-termini by an additional disulfide bond. This solution for extreme stability was also found in the spider toxin ω-hexatoxin-Hv1a. This innovation in protein stability is beneficial for a protein design approach for a biochemical stable scaffold [48].

Short versions of the AMP peptide, with three disulfide bonds resembling defensin were identified in marine sponges [73] and jellyfish [74]. In jellyfish, a similarity to defensin is extended also to the K^+^ ICIs of sea anemones. Multiple functionalities had been experimentally validated for the short CSαβ scaffold of DRS, and the truncated scorpion toxin. Both peptides are effective as ion channel modulators (on *D. melanogaster* voltage-gated sodium channel) and exhibit anti-fungal activity [75].

### 2.3. iTOLIPs as Ion Channel Inhibitors

We analyzed proteins whose structural similarity to toxins have been identified. Table 2 lists nine instances in which a toxin related function is revealed. All 9 proteins exhibit channel blocker similarity to various channels [76]. Interestingly, two sequences from the *Apis mellifera* (Honeybee) and *Aphidius ervi* (Aphid parasite) show a clear homology to ω-conotoxin MVIIC and GVIA, a potent conus peptide that effectively blocks Ca^2+^ channels. The OCLP1 was initially identified using ClanTox, and its function as ICI had been validated [11].

We retested the OCLP1 structural model in view of the doubling of proteins with 3D-structures in the last decade. The most likely structural model for OCLP1 benefited from structural relatedness (Figure 4). The similarity in the cysteine distribution locations along the sequence, and the cysteines that contribute to the disulfide bridges applies for ω-conotoxin MVIIC (1cnn.1, 1omn.1), Ptu-1 (1i26.1), Toxin Ado1 (1lmr. 1), SVIB (1mvj.1), ω-conotoxin GVIA (1omc.1, 1tr6.1, 1ttl.1, 2cco.1), Robustoxin (1qdp.1), Hainantoxin-3 (2jtb.1), Spiderine-1a (2n86.1), and more. Importantly, the OCLP1 model indicates a comparable sequence similarity to a large number of ICIs. The related sequences exhibiting ICI function blocks Na^+^, K^+^, and all major types of Ca^+2^ channels (L-, N-, and P/Q-types, Figure 4). As such, these sequences are attractive templates for drug development seeking feature determinants that dictate a detailed specificity. Actually, the specificity is not restricted to the selective ion but to the exact version of the ion channel. For example, the protein μ-theraphotoxin-Pn3a that was isolated from venom of the tarantula *Pamphobeteus nigricolor,* is a potent inhibitor of Nav1.7, a subtype of the sodium ion channel (Nav). Its specificity for the other Nav subtypes is lower by 2–3 order of magnitudes [77].

A detailed report for the five top templates that are used for construction of a structural model for each of the 9 proteins (Table 2) is available (Table S2).

### 2.4. Uncharacterized iTOLIPs Reveal New Cysteine-Rich Patterns 

**Figure 5:**
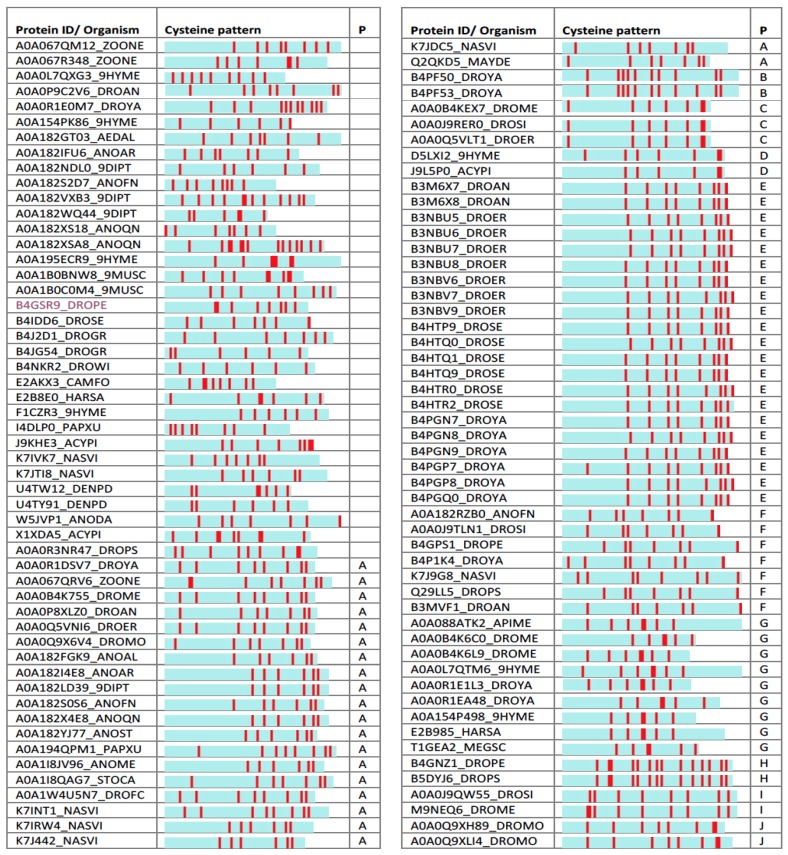
**Uncharacterized iTOLIPs and a graphical representations of the mini-proteins**. The cysteine residues are marked by red bars. The proteins are grouped according to the recurrent pattern of cysteines based on their number and location along the protein sequence (P, pattern).

Among the identified mini-proteins are 110 sequences that are annotated as “uncharacterized” (and genes named by their genomic index). About 65% of them are from Diptera (55 from Drosophilae, and 16 from Anopheles). Inspecting the spacing and number of the cysteines among the “uncharacterized” mini-proteins shows numerous recurring patterns (Figure 5).

**Figure 6:**
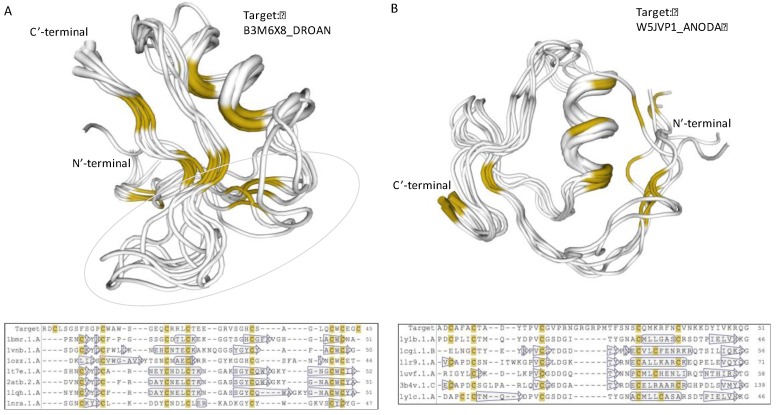
**Structural model of iTOLIPs uncharacterized proteins**. (**A**) Structural model of the protein B3M6X8_DROAN is shown. The structural model is a defensin fold. The overlap of 7 patterns is shown along with their multiple sequence alignments. The light green marks the area in which the sequences vary the most among the representative proteins. (**B**) Structural model of the protein W5JVP1_ANODA is shown. The structural model is of the Kazal protease inhibitor fold. The overlap of 6 patterns is shown along with their multiple sequence alignments. The positions of the α-helix and β-sheets are shown by the hollow frame and arrows, respectively. Yellow color marks the position of the cysteines.

A recurring pattern is illustrated by the B3M6X8_DROAN (*Drosophila ananassae*). This pattern is identified in *Drosophila erecta* and *Drosophila yabuba*, and appears in 20 proteins (with small variations, Figure 5, Patten E). Using structural modeling, we found that the strongest sequence similarity is to PDB: 1myn.1 (Drosomycin). Yet, another set of toxins such as the α-like toxin Lqh3 and BmαTX47 toxins from old and new world scorpions [78] seems to share a structural fold (Figure 6A). All these neurotoxins are specific to different Nav subtypes [79]. The stiff structure is visible mainly through the α-helix and the antiparallel β-sheets (Figure 6A). However, the substantial variations in the loops indicate the potential site for specificity of AMP, and the K^+^ and Na^+^ ion channel blocking. The overlap of B3M6X relative to 7 protein representatives that contributed to the model is shown along their multiple sequence alignment (Figure 6A, bottom).

A systematic search for a model for the uncharacterized proteins showed that for A0A182S0S6_ANOFN (*Anopheles funestus*, Figure 5, Patten A), the best model is similar to gamma 1-P thionins from barley and wheat endosperm (PDB: 1gps). These proteins are common motifs among toxic arthropod proteins and defensins. Still, the most likely defensin that was associated with *Anopheles funestus* protein is from a plant origin (PDB: 5nce.1).

Modeling the structure of the uncharacterized W5JVP1_ANODA (Figure 5, Pattern F) revealed a strong and highly conserved structure similar to a “non-classical” Kazal-type inhibitor (Figure 6B). All six structure representatives are aligned, and support its function as protease inhibitor. Kazal protease inhibitor fold was identified from some snakes, sea anemone, and skin of tree frogs. However, most proteinase inhibitor from toxins are associated with Kunitz fold that display a broader taxonomical coverage and a robust protease inhibition [80]. Other proteins predicted by structural modeling to have the Kazal protease inhibitor fold include A0A182RZB0_ANOFN, A0A0J9TLN1_DROSI, Q29LL5_DROPS, K7J9G8_NASVI, B3MVF1_DROAN, and B4GPS1_DROPE (Figure 5, Patten F).

Testing other uncharacterized proteins from the list (Figure 5) resulted in poor or no supportive models. Note that some cysteine-based patterns appear with multiple examples in the list. For example, B4PF50_DROYA and B4PF53_DROYA share the same pattern in terms of their cysteine number and spacing (Figure 5, Pattern B). Additional proteins are associated with structurally new shapes that could not be modeled to reach a satisfactory level (e.g., A0A0P9C2V6_DROAN). These findings suggest that the uncharacterized proteins provide a rich, yet unexplored scaffold for future drug design.

## 3. Materials and Methods 

### 3.1. Protein Databases 

We used datasets from UniProtKB Release Aug_2017 [81] including 90 million protein sequences, combining the SwissProt and TrEMBL datasets [82]. We used the current data from RCSB protein data bank [83] with the collection of about 124,000 proteins’ structural information.

### 3.2. Bioinformatics Analysis Tools

SignalP 4.0 was used to predict signal peptides [84]. This self-standing predictive tool is also provided as an annotation in UniProtKB [KW-0732]. The average length of the signal sequence in mammals is about 25 AA. We consider a protein length of 75 AA to account for a mature protein of about 50 AA. EBI’s ClustalW and alignment viewer tools were used. Swiss-Model [82] was applied with default parameters for building a model according to the templates from the RCSB database. In the automated mode, both BLAST and HHblits (profile -profile search) are used. HHpred and HHblits [85] provide sensitive structural prediction by HMM -HMM- comparison. The HHblits builds HMM from a query sequence and compares it with a library of HMMs representing all known structures from PDB [83]. All structural predictions obtained from Swiss-Model, and HHblits were compared for testing the quality of the results.

Template quality is estimated along the process of the model building, for maximization of the quality and coverage of the model. In some cases, more than one model is presented to reflect the structural diversity. The quality of the models is estimated using calculated statistical parameters of the model (GMQE and QMEAN). These values are determined with respect to experimental parameters of proteins with a similar length ([82]). Only sufficiently supported quality models are presented. The visualization tool used are embedded in Swiss-Model. A sequence similarity map shows the proteins that were used as templates, and contributed to the final model from a set of non-redundant structurally solved proteins.

### 3.3. ClanTox Prediction and Scoring

ClanTox (classifier of animal toxins) is a machine learning classifier ensemble for ranking protein sequences according to their toxin-like properties. ClanTox provides characterization for these mostly uncharacterized proteins. ClanTox uses about 600 features, including the stability and the spacing of the cysteine residues [57]. However, features are not restricted to cysteine-related features. ClanTox was trained on few hundreds of ICIs from a broad range of animal toxins. ClanTox’s method represents each sequence as a vector of numerical sequence-derived features. The test set performance of ClanTox in cross-validation is very high, with a mean area under the curve (AUC) of >0.99 [86].

The sequences from the selected subset of insect proteomes downloaded from UniProtKB were used as input for ClanTox. The classifier outputs four labels: N for negative prediction, and P1–P3, reflecting three levels of positive predictions for toxin-like proteins (TOLIPs). The most significant predictions (labeled P3) accounts for proteins with a mean score >0.2, as well as having a coefficient of variation (CV) <0.5. The negative predictions (N, predicted as non-toxin) account for all sequences with a mean score <−0.2. The confidence of the prediction indirectly considers the robustness of the prediction. Formally, P3 are predictions with a mean score >0.2 or mean score >2*SD; P2 are predictions with mean score >0.2 or mean score between SD and 2*SD; and P1 are predictions with mean score >−0.2 or mean score <SD [57].

## 4. Conclusions

From the evolutionary perspective, toxins that possess similar functions (e.g., ICIs) may appear in unrelated venomous species, which is in accord with an accelerated evolution and innovation among toxins. Detecting endogenous toxin-like proteins from insects (iTOLIPs) confirmed that much of the innovation associated with bioactive peptides and mini-proteins links to defense against microbes, mainly fungi, and modulating of ion channels. Potentially, these functions are not mutually exclusive, and short proteins may carry more than one function. The rich collection identified in insects is instrumental in searching for particular AA that can enhance specificity towards specific fungi, or bacterium in the case of AMPs. In this study, we discussed a collection of top predictions from ClanTox. Note that hundreds of additional iTOLIPs are reported at somewhat lower predicted confidence. We conclude that the overlooked iTOLIPs characterized by structural stability and enhanced specificity are attractive templates for drug design.

## Figures and Tables

**Table 1:** iTOLIPs top predictions by major insects’ order.

Insects	Number of Short Proteins	Number of Top Predictions	% Top Predictions from Total	Representative Family
Blattoidea	238	124	52.1	Termite
Hymenoptera (wasps, ants and bees)	460	35	7.6	Honeybee
Ditrysia	403	20	5	Butterfly
Polyphaga	139	12	8.6	Beetle
Hemiptera	230	16	7	Aphid
Pulicidae	17	2	11.8	Flea
Acrididae	9	2	22.2	Grasshopper
Pseudagrion	2	0	0	Damselfly
Psocodea	24	0	0	Lice
**All insects**	**4196**	**379**	**9**	

**Table 2:** Toxin-like mini-proteins from insects.

UniProtKB	AA (Mature) ^a^	Protein Name	Species	PDB	% Seq. Sim	Description
H9KQJ7	74 (54)	ω-conotoxin-like protein 1	*A. mellifera*	2n86.1	44.1	Spiderine-1a
A0A084WJA1	71 (46)	K-channel toxin α-KTx 18.3	*A. sinensis*	2b68.1	24.1	defensin
J7HBU2	70 (47)	Salivary toxin-like peptide	*N. intermedia*	5t4r.1	51.5	Mu-theraphotoxin-Pn3a
J7HIK0	70 (47)	Salivary toxin-like peptide	*N. intermedia*	5t4r.1	51.5	Mu-theraphotoxin-Pn3a
J7HBS6	70 (46)	Salivary toxin-like peptide	*N. intermedia*	5t4r.1	51.5	Mu-theraphotoxin-Pn3a
J7HBT1	75 (50)	Salivary toxin-like peptide	*N. intermedia*	1d1h.1	46.7	Hanatoxin Type 1
A0A034WXR3	60 (36)	Venom toxin-like peptide	*A. ervi*	1q3j.1	33.3	ALO3
A0A034WY34	61 (37)	Venom toxin-like peptide	*A. ervi*	2lqa.1	43.8	Asteropsin A
A0A034WWW1	51 (37)	Venom toxin-like peptide	*A. ervi*	1omn.1	48.0	ω-Conotoxin MVIIC

**^a^** Full length of the protein, and the length of the mature protein (in parentheses). Mature protein is a cleaved product after removal of the N’-terminal signal sequence. Seq. sim, sequence similarity.

## Supplementary Materials

The following are available online at www.mdpi.com/2072-6651/9/11/350/s1, Table S1: Top prediction of ClanTox (P3) for insect < 75 AA with 379 iTOLIPs. Table S2: Top 5 templates selected by Swiss-Model for constructing the structural model of nine mature iTOLIP mini-protein. Figure S1. Scoring of ClanTox predictions for insects’ secreted mini-proteins. Distribution of ClanTox predictions of 4180 insects’ secreted proteins shorter than 75 AA. The top scoring iTOLIPs are marked by P3 (dark red), the intermediate confidence is P2 and P1 is the least confident predictions. The gray marks the bulk of the sequences (76%) with negative prediction (i.e., not a TOLIPs). All together there are 379 proteins that are scored as P3 (Table S1).

## Abbreviations

AMP	antimicrobial peptides
CSαβ	cysteine-stabilized α-helical and β-sheet
ClanTox	classifier of animal toxins
CRISP	cysteine rich short proteins
ICI	ion channel inhibitor
DRS	Drosomycin
nAChR	nicotinic acetylcholine receptors
OCLP	omega conotoxin-like protein
TFP	three-finger proteins
iTOLIP	insect toxin-like proteins

## References

[1] Adermann K., John H., Standker L., Forssmann W.G. (2004). Exploiting natural peptide diversity: Novel research tools and drug leads. Curr. Opin. Biotechnol..

[2] Alonso D., Khalil Z., Satkunanthan N., Livett B.G. (2003). Drugs from the sea: Conotoxins as drug leads for neuropathic pain and other neurological conditions. Mini Rev. Med. Chem..

[3] King G.F. (2011). Venoms as a platform for human drugs: Translating toxins into therapeutics. Expert Opin. Biol. Ther..

[4] Proksch P., Edrada R., Ebel R. (2002). Drugs from the seas-current status and microbiological implications. Appl. Microbiol. Biotechnol..

[5] Bock J.E., Gavenonis J., Kritzer J.A. (2013). Getting in shape: Controlling peptide bioactivity and bioavailability using conformational constraints. ACS Chem. Biol..

[6] Vetter I., Davis J.L., Rash L.D., Anangi R., Mobli M., Alewood P.F., Lewis R.J., King G.F. (2011). Venomics: A new paradigm for natural products-based drug discovery. Amino Acids.

[7] Bulaj G. (2008). Integrating the discovery pipeline for novel compounds targeting ion channels. Curr. Opin. Chem. Biol..

[8] Harvey A.L. (2014). Toxins and drug discovery. Toxicon.

[9] Fry B.G., Roelants K., Champagne D.E., Scheib H., Tyndall J.D., King G.F., Nevalainen T.J., Norman J.A., Lewis R.J., Norton R.S. (2009). The toxicogenomic multiverse: Convergent recruitment of proteins into animal venoms. Annu. Rev. Genom. Hum. Genet..

[10] Wong E.S., Belov K. (2012). Venom evolution through gene duplications. Gene.

[11] Kaplan N., Morpurgo N., Linial M. (2007). Novel families of toxin-like peptides in insects and mammals: A computational approach. J. Mol. Biol..

[12] Fry B.G., Wuster W., Kini R.M., Brusic V., Khan A., Venkataraman D., Rooney A.P. (2003). Molecular evolution and phylogeny of elapid snake venom three-finger toxins. J. Mol. Evol..

[13] Craik D.J., Fairlie D.P., Liras S., Price D. (2013). The future of peptide-based drugs. Chem. Biol. Drug Des..

[14] Han T.S., Teichert R.W., Olivera B.M., Bulaj G. (2008). Conus venoms—A rich source of peptide-based therapeutics. Curr. Pharm. Des..

[15] Lavergne V., Harliwong I., Jones A., Miller D., Taft R.J., Alewood P.F. (2015). Optimized deep-targeted proteotranscriptomic profiling reveals unexplored conus toxin diversity and novel cysteine frameworks. Proc. Natl. Acad. Sci. USA.

[16] Drabeck D.H., Dean A.M., Jansa S.A. (2015). Why the honey badger don’t care: Convergent evolution of venom-targeted nicotinic acetylcholine receptors in mammals that survive venomous snake bites. Toxicon.

[17] Zambelli V., Pasqualoto K., Picolo G., Chudzinski-Tavassi A., Cury Y. (2016). Harnessing the knowledge of animal toxins to generate drugs. Pharmacol. Res..

[18] Fry B.G. (2005). From genome to “venome” Molecular origin and evolution of the snake venom proteome inferred from phylogenetic analysis of toxin sequences and related body proteins. Genome Res..

[19] Casewell N.R., Wuster W., Vonk F.J., Harrison R.A., Fry B.G. (2013). Complex cocktails: The evolutionary novelty of venoms. Trends Ecol. Evol..

[20] Sitprija V., Sitprija S. (2012). Renal effects and injury induced by animal toxins. Toxicon.

[21] Corzo G., Villegas E., Gomez-Lagunas F., Possani L.D., Belokoneva O.S., Nakajima T. (2002). Oxyopinins, large amphipathic peptides isolated from the venom of the wolf spider oxyopes kitabensis with cytolytic properties and positive insecticidal cooperativity with spider neurotoxins. J. Biol. Chem..

[22] Edwards L.P., Whitter E., Hessinger D.A. (2002). Apparent membrane pore-formation by portuguese man-of-war (physalia physalis) venom in intact cultured cells. Toxicon.

[23] Slotta K.H., Gonzalez J., Roth S. (2016). The direct and indirect hemolytic factors from animal venoms. RUSSELL Animal Toxins.

[24] Estrada G., Villegas E., Corzo G. (2007). Spider venoms: A rich source of acylpolyamines and peptides as new leads for cns drugs. Nat. Prod. Rep..

[25] Petricevich V.L. (2010). Scorpion venom and the inflammatory response. Mediat. Inflamm..

[26] Gibbons A., Dean B. (2016). The cholinergic system: An emerging drug target for schizophrenia. Curr. Pharm. Des..

[27] Tirosh Y., Ofer D., Eliyahu T., Linial M. (2013). Short toxin-like proteins attack the defense line of innate immunity. Toxins.

[28] Tsetlin V.I. (2015). Three-finger snake neurotoxins and ly6 proteins targeting nicotinic acetylcholine receptors: Pharmacological tools and endogenous modulators. Trends Pharmacol. Sci..

[29] Kini R.M. (2011). Evolution of three-finger toxins—A versatile mini protein scaffold. Acta Chim. Slovenica.

[30] Ibanez-Tallon I., Miwa J.M., Wang H.L., Adams N.C., Crabtree G.W., Sine S.M., Heintz N. (2002). Novel modulation of neuronal nicotinic acetylcholine receptors by association with the endogenous prototoxin lynx1. Neuron.

[31] Chimienti F., Hogg R.C., Plantard L., Lehmann C., Brakch N., Fischer J., Huber M., Bertrand D., Hohl D. (2003). Identification of slurp-1 as an epidermal neuromodulator explains the clinical phenotype of mal de meleda. Hum. Mol. Genet..

[32] Kalia J., Milescu M., Salvatierra J., Wagner J., Klint J.K., King G.F., Olivera B.M., Bosmans F. (2015). From foe to friend: Using animal toxins to investigate ion channel function. J. Mol. Biol..

[33] Mouhat S., Andreotti N., Jouirou B., Sabatier J.-M. (2008). Animal toxins acting on voltage-gated potassium channels. Curr. Pharm. Des..

[34] Norton R.S. (1998). Structure and function of peptide and protein toxins from marine organisms. J. Toxicol. Toxin Rev..

[35] Terlau H., Olivera B.M. (2004). Conus venoms: A rich source of novel ion channel-targeted peptides. Physiol. Rev..

[36] Quintero-Hernández V., Jiménez-Vargas J., Gurrola G., Valdivia H., Possani L. (2013). Scorpion venom components that affect ion-channels function. Toxicon.

[37] Bohlen C.J., Chesler A.T., Sharif-Naeini R., Medzihradszky K.F., Zhou S., King D., Sánchez E.E., Burlingame A.L., Basbaum A.I., Julius D. (2011). A heteromeric texas coral snake toxin targets acid-sensing ion channels to produce pain. Nature.

[38] Guo M., Teng M., Niu L., Liu Q., Huang Q., Hao Q. (2005). Crystal structure of the cysteine-rich secretory protein stecrisp reveals that the cysteine-rich domain has a K^+^ channel inhibitor-like fold. J. Biol. Chem..

[39] Gibbs G.M., Orta G., Reddy T., Koppers A.J., Martínez-López P., de la Vega-Beltràn J.L., Lo J.C., Veldhuis N., Jamsai D., McIntyre P. (2011). Cysteine-rich secretory protein 4 is an inhibitor of transient receptor potential m8 with a role in establishing sperm function. Proc. Natl. Acad. Sci. USA.

[40] Diochot S., Salinas M., Baron A., Escoubas P., Lazdunski M. (2007). Peptides inhibitors of acid-sensing ion channels. Toxicon.

[41] Mouhat S., Jouirou B., Mosbah A., De Waard M., Sabatier J.-M. (2004). Diversity of folds in animal toxins acting on ion channels. Biochem. J..

[42] Ohno M., Menez R., Ogawa T., Danse J.M., Shimohigashi Y., Fromen C., Ducancel F., Zinn-Justin S., Le Du M.H., Boulain J.C. (1998). Molecular evolution of snake toxins: Is the functional diversity of snake toxins associated with a mechanism of accelerated evolution?. Prog. Nucl. Acid Res. Mol. Biol..

[43] Chang L.-S. (2007). Genetic diversity in snake venom three-finger proteins and phospholipase a2 enzymes. Toxin Rev..

[44] Casewell N.R., Wagstaff S.C., Harrison R.A., Renjifo C., Wüster W. (2011). Domain loss facilitates accelerated evolution and neofunctionalization of duplicate snake venom metalloproteinase toxin genes. Mol. Biol. Evol..

[45] Banerjee A., Lee A., Campbell E., MacKinnon R. (2013). Structure of a pore-blocking toxin in complex with a eukaryotic voltage-dependent K^+^ channel. Elife.

[46] Strix G. (2005). A toxin against pain. Sci. Am..

[47] Góngora-Benítez M., Tulla-Puche J., Albericio F. (2013). Multifaceted roles of disulfide bonds. Peptides as therapeutics. Chem. Rev..

[48] Herzig V., King G.F. (2015). The cystine knot is responsible for the exceptional stability of the insecticidal spider toxin ω-hexatoxin-hv1a. Toxins.

[49] Kuzmenkov A.I., Fedorova I.M., Vassilevski A.A., Grishin E.V. (2013). Cysteine-rich toxins from lachesana tarabaevi spider venom with amphiphilic c-terminal segments. Biochim. Biophys. Acta.

[50] Lavergne V., Alewood P.F., Mobli M., King G.F. (2015). The structural universe of disulfide-rich venom peptides. Venoms to Drugs: Venoms as a Source for the Development of Human Therapeutics.

[51] Avrutina O. (2016). Synthetic cystine-knot miniproteins—Valuable scaffolds for polypeptide engineering. Adv. Exp. Med. Biol..

[52] Rappoport N., Karsenty S., Stern A., Linial N., Linial M. (2012). Protonet 6.0: Organizing 10 million protein sequences in a compact hierarchical family tree. Nucl. Acids Res..

[53] Ofer D., Rappoport N., Linial M. (2015). The little known universe of short proteins in insects: A machine learning approach. Short Views on Insect Genomics and Proteomics.

[54] Werren J.H., Richards S., Desjardins C.A., Niehuis O., Gadau J., Colbourne J.K., Group N.G.W. (2010). Functional and evolutionary insights from the genomes of three parasitoid nasonia species. Science.

[55] Nygaard S., Zhang G., Schiøtt M., Li C., Wurm Y., Hu H., Zhou J., Ji L., Qiu F., Rasmussen M. (2011). The genome of the leaf-cutting ant acromyrmex echinatior suggests key adaptations to advanced social life and fungus farming. Genome Res..

[56] Rappoport N., Linial M. (2015). Trends in genome dynamics among major orders of insects revealed through variations in protein families. BMC Genom..

[57] Naamati G., Askenazi M., Linial M. (2009). Clantox: A classifier of short animal toxins. Nucleic Acids Res..

[58] Radivojac P., Clark W.T., Oron T.R., Schnoes A.M., Wittkop T., Sokolov A., Graim K., Funk C., Verspoor K., Ben-Hur A. (2013). A large-scale evaluation of computational protein function prediction. Nat. Methods.

[59] Kaplan N., Linial M. (2005). Automatic detection of false annotations via binary property clustering. BMC Bioinform..

[60] Ofer D., Linial M. (2013). Neuropid: A predictor for identifying neuropeptide precursors from metazoan proteomes. Bioinformatics.

[61] Tirosh Y., Linial I., Askenazi M., Linial M. (2012). Short toxin-like proteins abound in cnidaria genomes. Toxins.

[62] Tassanakajon A., Somboonwiwat K., Amparyup P. (2015). Sequence diversity and evolution of antimicrobial peptides in invertebrates. Dev. Comp. Immunol..

[63] Liu Z., Yuan K., Zhang R., Ren X., Liu X., Zhao S., Wang D. (2016). Cloning and purification of the first termicin-like peptide from the cockroach eupolyphaga sinensis. J. Venom. Anim. Toxins Incl. Trop. Dis..

[64] Fjell C.D., Hiss J.A., Hancock R.E., Schneider G. (2011). Designing antimicrobial peptides: Form follows function. Nat. Rev. Drug Discov..

[65] Froy O., Gurevitz M. (2004). Arthropod defensins illuminate the divergence of scorpion neurotoxins. J. Pept. Sci..

[66] Froy O., Gurevitz M. (2003). New insight on scorpion divergence inferred from comparative analysis of toxin structure, pharmacology and distribution. Toxicon.

[67] Bun Ng T., Chi Fai Cheung R., Ho Wong J., Juan Ye X. (2013). Antimicrobial activity of defensins and defensin-like peptides with special emphasis on those from fungi and invertebrate animals. Curr. Protein Pept. Sci..

[68] Whittington C.M., Papenfuss A.T., Bansal P., Torres A.M., Wong E.S., Deakin J.E., Graves T., Alsop A., Schatzkamer K., Kremitzki C. (2008). Defensins and the convergent evolution of platypus and reptile venom genes. Genome Res..

[69] Varkey J., Singh S., Nagaraj R. (2006). Antibacterial activity of linear peptides spanning the carboxy-terminal beta-sheet domain of arthropod defensins. Peptides.

[70] Zhu S., Li W., Jiang D., Zeng X. (2000). Evidence for the existence of insect defensin-like peptide in scorpion venom. IUBMB Life.

[71] Gao B., Zhu S. (2016). The drosomycin multigene family: Three-disulfide variants from drosophila takahashii possess antibacterial activity. Sci. Rep..

[72] Deng X.J., Yang W.Y., Huang Y.D., Cao Y., Wen S.Y., Xia Q.Y., Xu P. (2009). Gene expression divergence and evolutionary analysis of the drosomycin gene family in drosophila melanogaster. J. Biomed. Biotechnol..

[73] Li H., Su M., Hamann M.T., Bowling J.J., Kim H.S., Jung J.H. (2014). Solution structure of a sponge-derived cystine knot peptide and its notable stability. J. Nat. Prod..

[74] Ovchinnikova T.V., Balandin S.V., Aleshina G.M., Tagaev A.A., Leonova Y.F., Krasnodembsky E.D., Men’shenin A.V., Kokryakov V.N. (2006). Aurelin, a novel antimicrobial peptide from jellyfish aurelia aurita with structural features of defensins and channel-blocking toxins. Biochem. Biophys. Res. Commun..

[75] Cohen L., Moran Y., Sharon A., Segal D., Gordon D., Gurevitz M. (2009). Drosomycin, an innate immunity peptide of drosophila melanogaster, interacts with the fly voltage-gated sodium channel. J. Biol. Chem..

[76] Stehling E.G., Sforca M.L., Zanchin N.I., Oyama S., Pignatelli A., Belluzzi O., Polverini E., Corsini R., Spisni A., Pertinhez T.A. (2012). Looking over toxin-k(+) channel interactions. Clues from the structural and functional characterization of alpha-ktx toxin tc32, a kv1.3 channel blocker. Biochemistry.

[77] Deuis J.R., Dekan Z., Wingerd J.S., Smith J.J., Munasinghe N.R., Bhola R.F., Imlach W.L., Herzig V., Armstrong D.A., Rosengren K.J. (2017). Pharmacological characterisation of the highly nav1.7 selective spider venom peptide pn3a. Sci. Rep..

[78] Jablonsky M.J., Jackson P.L., Krishna N.R. (2001). Solution structure of an insect-specific neurotoxin from the new world scorpion centruroides sculpturatus ewing. Biochemistry.

[79] Krimm I., Gilles N., Sautiere P., Stankiewicz M., Pelhate M., Gordon D., Lancelin J.M. (1999). Nmr structures and activity of a novel alpha-like toxin from the scorpion leiurus quinquestriatus hebraeus. J. Mol. Biol..

[80] Mourao C.B., Schwartz E.F. (2013). Protease inhibitors from marine venomous animals and their counterparts in terrestrial venomous animals. Mar. Drugs.

[81] Boutet E., Lieberherr D., Tognolli M., Schneider M., Bansal P., Bridge A.J., Poux S., Bougueleret L., Xenarios I. (2016). Uniprotkb/swiss-prot, the manually annotated section of the uniprot knowledgebase: How to use the entry view. Plant Bioinformatics: Methods and Protocols.

[82] Bienert S., Waterhouse A., de Beer T.A., Tauriello G., Studer G., Bordoli L., Schwede T. (2017). The swiss-model repository-new features and functionality. Nucleic Acids Res..

[83] Rose P.W., Prlić A., Altunkaya A., Bi C., Bradley A.R., Christie C.H., Costanzo L.D., Duarte J.M., Dutta S., Feng Z. (2016). The rcsb protein data bank: Integrative view of protein, gene and 3d structural information. Nucleic Acids Res..

[84] Petersen T.N., Brunak S., von Heijne G., Nielsen H. (2011). Signalp 4.0: Discriminating signal peptides from transmembrane regions. Nat. Methods.

[85] Remmert M., Biegert A., Hauser A., Söding J. (2012). Hhblits: Lightning-fast iterative protein sequence searching by hmm-hmm alignment. Nat. Methods.

[86] Naamati G., Askenazi M., Linial M. (2010). A predictor for toxin-like proteins exposes cell modulator candidates within viral genomes. Bioinformatics.

