# Supplementary material for: Overlooked Short Toxin-Like Proteins: A Shortcut to Drug Design
Source: Toxins (Basel). 2017 Oct 29;9(11):350. doi: 10.3390/toxins9110350 (PMC5705965; doi:10.3390/toxins9110350)
Supplement: Supplementary file 1 [file toxins-09-00350-s001.docx]

**Supplementary Materials: Overlooked Short Toxin-like Proteins: A Shortcut to Drug Design**

Michal Linial, Nadav Rappoport and Dan Ofer

**Table S1.** Top prediction of ClanTox (P3) for insect < 75 AA with 379 iTOLIPs

| Entry Name | Protein Names | Organism | Length |
| --- | --- | --- | --- |
| Q174X9_AEDAE | AAEL006746-PA (Defensin anti-microbial peptide) | Aedes aegypti (Yellowfever mosquito) (Culex aegypti) | 74 |
| Q20DK8_DROTE | ACP158 | Drosophila teissieri (Fruit fly) | 71 |
| Q2VKC8_DROAR | Acp25 | Drosophila arizonae (Fruit fly) | 43 |
| Q2VKC9_DROAR | Acp25 | Drosophila arizonae (Fruit fly) | 43 |
| Q2VKD2_DROAR | Acp25 | Drosophila arizonae (Fruit fly) | 43 |
| Q2VKC3_DROMO | Acp25 | Drosophila mojavensis (Fruit fly) | 43 |
| Q2VKC2_DROMO | Acp25 | Drosophila mojavensis (Fruit fly) | 43 |
| Q2VKC1_DROMO | Acp25 | Drosophila mojavensis (Fruit fly) | 43 |
| Q38RA0_DROSI | ACP54A1 | Drosophila simulans (Fruit fly) | 46 |
| Q45WI3_DROSI | ACP54A1 | Drosophila simulans (Fruit fly) | 46 |
| A7UUD8_ANOGA | AGAP006568-PA | Anopheles gambiae (African malaria mosquito) | 68 |
| A0A084WMQ0_ANOSI | AGAP006568-PA-like protein | Anopheles sinensis (Mosquito) | 61 |
| APAM_APICC | Apamin | Apis cerana cerana (Oriental honeybee) | 46 |
| APAM_APIME | Apamin | Apis mellifera (Honeybee) | 46 |
| B7UUK0_APIME | Apamin protein | Apis mellifera (Honeybee) | 46 |
| A0A1N7TCE5_AGRPL | Aplamycin-5 | Agrilus planipennis (Emerald ash borer) (Agrilus marcopoli) | 66 |
| A0A1N7TCE4_AGRPL | Aplamycin-5 (drosomycin-like isoform X3) | Agrilus planipennis (Emerald ash borer) (Agrilus marcopoli) | 66 |
| A0A1N7TCE6_AGRPL | Aplamycin-6 (drosomycin-like) | Agrilus planipennis (Emerald ash borer) (Agrilus marcopoli) | 66 |
| X5CH30_CALMS | Callomycin-1 | Callosobruchus maculatus (Southern cowpea weevil) (Pulse bruchid) | 68 |
| A0A0M4ECI6_DROBS | CG42486 | Drosophila busckii (Fruit fly) | 73 |
| A0A1S3DVV4_DIACI | chorion class high-cysteine HCB protein 13-like | Diaphorina citri (Asian citrus psyllid) | 74 |
| A0A1J1IFI4_9DIPT | CLUMA_CG012096, isoform A | Clunio marinus | 66 |
| C6K2I0_BOMMO | Cold-related protein | Bombyx mori (Silk moth) | 73 |
| R4WD45_RIPPE | Cysteine rich secreted protein | Riptortus pedestris (Bean bug) | 61 |
| R4WE54_RIPPE | Cysteine rich secreted protein | Riptortus pedestris (Bean bug) | 68 |
| R4WDC6_RIPPE | Cysteine rich secreted protein | Riptortus pedestris (Bean bug) | 71 |
| R4WNR0_RIPPE | Cysteine rich secreted protein | Riptortus pedestris (Bean bug) | 68 |
| R4WIX2_RIPPE | Cysteine rich secreted protein | Riptortus pedestris (Bean bug) | 72 |
| R4WDU9_RIPPE | Cysteine rich secreted protein | Riptortus pedestris (Bean bug) | 64 |
| CVP3_PIMHY | Cysteine-rich venom protein 3 (cvp3) | Pimpla hypochondriaca (Parasitoid wasp) | 63 |
| A0A1W4WJ82_AGRPL | defense protein 6-like | Agrilus planipennis (Emerald ash borer) (Agrilus marcopoli) | 69 |
| R4IXW7_BEMTA | Defensin | Bemisia tabaci (Sweetpotato whitefly) (Aleurodes tabaci) | 75 |
| B6DE47_ANODA | Defensin | Anopheles darlingi (Mosquito) | 57 |
| Q95UJ8_PYRRU | Defensin | Pyrocoelia rufa (Firefly) | 55 |
| DEF1_GALME | Defensin (Galiomicin) | Galleria mellonella (Greater wax moth) | 72 |
| E5LBK2_MANSE | Defensin 2 | Manduca sexta (Tobacco hawkmoth) (Tobacco hornworm) | 70 |
| V9MMW2_COTVE | Defensin 2 (T-defensin-2) | Cotesia vestalis (Diamondback moth parasite) (Cotesia plutellae) | 73 |
| V9MG79_COTVE | Defensin 3 | Cotesia vestalis (Diamondback moth parasite) (Cotesia plutellae) | 73 |
| A0A140IM59_LOCMI | Defensin 5 | Locusta migratoria (Migratory locust) | 67 |
| B0WXK9_CULQU | Defensin anti-microbial peptide | Culex quinquefasciatus (Southern house mosquito) (Culex pungens) | 75 |
| R4WHS9_RIPPE | Defensin like protein | Riptortus pedestris (Bean bug) | 73 |
| C1IBY5_TABYA | Defensin TY 1 | Tabanus yao (Horsefly) | 69 |
| DIAP_GASAT | Diapause-specific peptide (DSP) (Diapausin) | Gastrophysa atrocyanea (Leaf beetle) | 65 |
| B3F883_SPOLT | Diapausin | Spodoptera litura (Asian cotton leafworm) | 63 |
| E1B227_SPOEX | Diapausin (Diapausin A2) (Diapausin A3) (Diapausin A5) (Diapausin A6) (Diapausin A7) | Spodoptera exigua (Beet armyworm) (Noctua fulgens) | 63 |
| A0A0G3DJA2_SPOEX | Diapausin A1 | Spodoptera exigua (Beet armyworm) (Noctua fulgens) | 63 |
| A0A0G3DRZ2_SPOEX | Diapausin A4 | Spodoptera exigua (Beet armyworm) (Noctua fulgens) | 63 |
| A0A0G3DJN3_SPOEX | Diapausin B1 | Spodoptera exigua (Beet armyworm) (Noctua fulgens) | 63 |
| Q2UYT3_DROSI | Dro1 protein | Drosophila simulans (Fruit fly) | 70 |
| Q2UYJ8_DROME | Dro1 protein | Drosophila melanogaster (Fruit fly) | 69 |
| Q2UY31_DROME | Dro1 protein | Drosophila melanogaster (Fruit fly) | 70 |
| Q2UY02_DROSI | Dro1 protein (Drosomycin-1) | Drosophila simulans (Fruit fly) | 70 |
| Q9VZQ5_DROME | Dro1 protein (Drosomycin-like 1) (Drosomycin-like C1) (GEO11745p1) (RT02921p) | Drosophila melanogaster (Fruit fly) | 69 |
| Q2UYU5_DROSI | Dro2 protein | Drosophila simulans (Fruit fly) | 70 |
| Q2UYB4_DROME | Dro2 protein | Drosophila melanogaster (Fruit fly) | 70 |
| Q7YXH9_DROME | Dro2 protein (Drosomycin 2) (Drosomycin-like 2) (Drosomycin-like D) (Drosomycin-like H) | Drosophila melanogaster (Fruit fly) | 70 |
| Q2UYT7_DROSI | Dro3 protein | Drosophila simulans (Fruit fly) | 71 |
| Q2UY99_DROSI | Dro3 protein | Drosophila simulans (Fruit fly) | 71 |
| Q2UYQ9_DROSI | Dro3 protein | Drosophila simulans (Fruit fly) | 71 |
| Q2UY34_DROME | Dro3 protein | Drosophila melanogaster (Fruit fly) | 71 |
| Q2UYD4_DROME | Dro3 protein | Drosophila melanogaster (Fruit fly) | 71 |
| Q2UYM9_DROME | Dro3 protein | Drosophila melanogaster (Fruit fly) | 71 |
| Q2UYC0_DROME | Dro3 protein | Drosophila melanogaster (Fruit fly) | 71 |
| Q2UYI2_DROME | Dro3 protein | Drosophila melanogaster (Fruit fly) | 71 |
| Q2UYH5_DROME | Dro3 protein | Drosophila melanogaster (Fruit fly) | 71 |
| Q2UYI9_DROME | Dro3 protein | Drosophila melanogaster (Fruit fly) | 71 |
| Q2UY86_DROSI | Dro3 protein (Drosomycin-3) | Drosophila simulans (Fruit fly) | 71 |
| Q2UYU3_DROSI | Dro4 protein | Drosophila simulans (Fruit fly) | 71 |
| Q2UY39_DROSI | Dro4 protein | Drosophila simulans (Fruit fly) | 71 |
| Q8IRD6_DROME | Dro4 protein (Drosomycin 4) (Drosomycin-like 4) (Drosomycin-like F) | Drosophila melanogaster (Fruit fly) | 71 |
| Q2UYS7_DROSI | Dro4 protein (Drosomycin-4) | Drosophila simulans (Fruit fly) | 71 |
| Q2UY63_DROSI | Dro6 protein | Drosophila simulans (Fruit fly) | 72 |
| Q2UY76_DROSI | Dro6 protein | Drosophila simulans (Fruit fly) | 72 |
| Q2UY30_DROME | Dro6 protein | Drosophila melanogaster (Fruit fly) | 72 |
| Q2UYT2_DROSI | Dro6 protein (Drosomycin-6) | Drosophila simulans (Fruit fly) | 72 |
| Q9VZQ4_DROME | Dro6 protein (Drosomycin-like 6) (Drosomycin-like D) (Drosomycin-like I) (GEO02559p1) | Drosophila melanogaster (Fruit fly) | 72 |
| A0A1W4W0N6_DROFC | drosomycin | Drosophila ficusphila (Fruit fly) | 70 |
| DMYC_DROME | Drosomycin (Cysteine-rich peptide) | Drosophila melanogaster (Fruit fly) | 70 |
| Q2UYR8_DROSI | Drosomycin (Drs protein) | Drosophila simulans (Fruit fly) | 70 |
| B0LUZ1_DROME | Drosomycin 3 | Drosophila melanogaster (Fruit fly) | 71 |
| B0LUZ3_DROME | Drosomycin 5 | Drosophila melanogaster (Fruit fly) | 69 |
| B4QPI1_DROSI | Drosomycin-1 | Drosophila simulans (Fruit fly) | 69 |
| A0A0J9RN31_DROSI | Drosomycin-2 | Drosophila simulans (Fruit fly) | 70 |
| B4QPH3_DROSI | Drosomycin-5 | Drosophila simulans (Fruit fly) | 69 |
| A0A1W4VNJ3_DROFC | drosomycin-like | Drosophila ficusphila (Fruit fly) | 70 |
| A0A1W4VNB5_DROFC | drosomycin-like | Drosophila ficusphila (Fruit fly) | 69 |
| A0A1W4VZY1_DROFC | drosomycin-like | Drosophila ficusphila (Fruit fly) | 69 |
| A0A1W4XC81_AGRPL | drosomycin-like | Agrilus planipennis (Emerald ash borer) (Agrilus marcopoli) | 66 |
| Q8IRD7_DROME | Drosomycin-like 3 (Drosomycin-like E) (Drosomycin-like G) (GEO11147p1) | Drosophila melanogaster (Fruit fly) | 71 |
| Q9VZR2_DROME | Drosomycin-like 5 (Drosomycin-like E) (Drosomycin-like G) (GEO10021p1) | Drosophila melanogaster (Fruit fly) | 69 |
| Q95NH5_9MUSC | Drosomycin-like A (Drosomycin-like B) | Drosophila triauraria | 71 |
| A0A1W4XCF3_AGRPL | drosomycin-like isoform X1 | Agrilus planipennis (Emerald ash borer) (Agrilus marcopoli) | 66 |
| A0A1W4X1M0_AGRPL | drosomycin-like isoform X2 | Agrilus planipennis (Emerald ash borer) (Agrilus marcopoli) | 66 |
| Q2UY87_DROSI | Drs protein | Drosophila simulans (Fruit fly) | 70 |
| Q2UYT9_DROSI | Drs protein | Drosophila simulans (Fruit fly) | 70 |
| Q2UYR5_DROSI | Drs protein | Drosophila simulans (Fruit fly) | 70 |
| A0A0A6ZH91_DROTK | DRS-1 | Drosophila takahashii (Fruit fly) | 70 |
| A0A0A6ZHX4_DROTK | DRS-11-1 | Drosophila takahashii (Fruit fly) | 69 |
| A0A0A6ZHE3_DROTK | DRS-11-2 | Drosophila takahashii (Fruit fly) | 69 |
| A0A0A6ZH96_DROTK | DRS-11a (DRS-11b) (DRS-11c) | Drosophila takahashii (Fruit fly) | 69 |
| A0A0A6ZHE0_DROTK | DRS-11d | Drosophila takahashii (Fruit fly) | 69 |
| A0A0A6ZHE9_DROTK | DRS-1a | Drosophila takahashii (Fruit fly) | 70 |
| A0A0A6ZHR1_DROTK | DRS-1b | Drosophila takahashii (Fruit fly) | 70 |
| A0A0A6ZHW6_DROTK | DRS-2-1 | Drosophila takahashii (Fruit fly) | 69 |
| A0A0A6ZHR6_DROTK | DRS-4 (DRS-4a) | Drosophila takahashii (Fruit fly) | 70 |
| A0A0A6ZHF2_DROTK | DRS-6a (DRS-6c) | Drosophila takahashii (Fruit fly) | 72 |
| A0A0A6ZH94_DROTK | DRS-6b | Drosophila takahashii (Fruit fly) | 72 |
| E5D602_HELAM | Galiomicin | Helicoverpa armigera (Cotton bollworm) (Heliothis armigera) | 73 |
| B4JJY9_DROGR | GH12150 | Drosophila grimshawi (Fruit fly) (Idiomyia grimshawi) | 53 |
| B4J2D1_DROGR | GH15460 | Drosophila grimshawi (Fruit fly) (Idiomyia grimshawi) | 72 |
| B4JG54_DROGR | GH18176 | Drosophila grimshawi (Fruit fly) (Idiomyia grimshawi) | 61 |
| B4GNZ1_DROPE | GL13781 | Drosophila persimilis (Fruit fly) | 71 |
| B4GPS1_DROPE | GL15367 | Drosophila persimilis (Fruit fly) | 74 |
| B4GSR9_DROPE | GL26589 | Drosophila persimilis (Fruit fly) | 61 |
| B4HTR0_DROSE | GM14054 | Drosophila sechellia (Fruit fly) | 72 |
| B4HTQ9_DROSE | GM14055 | Drosophila sechellia (Fruit fly) | 69 |
| B4HTP9_DROSE | GM14560 | Drosophila sechellia (Fruit fly) | 70 |
| B4HTQ0_DROSE | GM14561 | Drosophila sechellia (Fruit fly) | 71 |
| B4HTQ1_DROSE | GM14562 | Drosophila sechellia (Fruit fly) | 69 |
| B4HTR2_DROSE | GM14569 | Drosophila sechellia (Fruit fly) | 72 |
| B4IDD6_DROSE | GM19432 | Drosophila sechellia (Fruit fly) | 62 |
| D3G9G5_HELVI | Heliomicin | Heliothis virescens (Tobacco budworm moth) | 73 |
| A0A109XLL6_BOMIG | Inhibitor cysteine knot | Bombus ignitus (Bumblebee) | 75 |
| V9IFQ3_APICE | Inhibitor cysteine knot peptide | Apis cerana (Indian honeybee) | 74 |
| A9XXB4_TRINI | Kalikludin | Trichoplusia ni (Cabbage looper) | 75 |
| A0A1B1V3G6_9DIPT | LolToxB | Bichromomyia olmeca | 75 |
| A0A1B1V3H1_9DIPT | LolToxI | Bichromomyia olmeca | 68 |
| V9I7A7_APICE | Low density lipoprotein receptor | Apis cerana (Indian honeybee) | 70 |
| MCDP_APIME | Mast cell degranulating peptide (MCD peptide) (MCDP) (Peptide 401) | Apis mellifera (Honeybee) | 50 |
| D3DMN2_DROME | MIP16286p | Drosophila melanogaster (Fruit fly) | 70 |
| OCLP1_APIME | Omega-conotoxin-like protein 1 (OCLP1) | Apis mellifera (Honeybee) | 74 |
| A0A084WJA1_ANOSI | Potassium channel toxin alpha-KTx 18.3 | Anopheles sinensis (Mosquito) | 71 |
| A5WYF3_STOCA | Protease inhibitor | Stomoxys calcitrans (Stable fly) (Conops calcitrans) | 72 |
| Q5MGE9_LONON | Protease inhibitor 9 | Lonomia obliqua (Moth) | 56 |
| Q0Q009_ANTMY | Protease inhibitor-like protein | Antheraea mylitta (Tasar silkworm) | 63 |
| Q0Q008_ANTMY | Protease inhibitor-like protein | Antheraea mylitta (Tasar silkworm) | 72 |
| A0A0M3VH87_SAMRI | Protease inhibitor1 | Samia ricini (Indian eri silkmoth) (Samia cynthia ricini) | 63 |
| Q2PQD0_BEMTA | Putative antimicrobial knottin protein Btk-1 | Bemisia tabaci (Sweetpotato whitefly) (Aleurodes tabaci) | 65 |
| Q2PQC7_BEMTA | Putative antimicrobial knottin protein Btk-4 | Bemisia tabaci (Sweetpotato whitefly) (Aleurodes tabaci) | 62 |
| A0A1L8E760_HAEIR | Putative kazal type serine protease inhibitor | Haematobia irritans (Horn fly) (Conops irritans) | 71 |
| R4G7P1_RHOPR | Putative kazal-type serine protease inhibitor | Rhodnius prolixus (Triatomid bug) | 73 |
| A0A023ED53_AEDAL | Putative knottins | Aedes albopictus (Asian tiger mosquito) (Stegomyia albopicta) | 73 |
| A0A023FBE8_TRIIF | Putative salivary kazaltype serine protease inhibitor | Triatoma infestans (Assassin bug) | 75 |
| A2IAA6_9NEOP | Putative secreted salivary protein | Xenopsylla cheopis (oriental rat flea) | 74 |
| A2IAD0_9NEOP | Putative secreted salivary protein | Xenopsylla cheopis (oriental rat flea) | 73 |
| Q3LTD6_9DIPT | Salivary gland antimicrobial peptide 1 | Bradysia hygida | 64 |
| B3NGW1_DROER | Salivary gland secretion 8 | Drosophila erecta (Fruit fly) | 75 |
| J7HBU2_9DIPT | Salivary toxin-like peptide | Nyssomyia intermedia | 70 |
| J7HIK0_9DIPT | Salivary toxin-like peptide | Nyssomyia intermedia | 70 |
| J7HBS6_9DIPT | Salivary toxin-like peptide | Nyssomyia intermedia | 70 |
| J7HBT1_9DIPT | Salivary toxin-like peptide | Nyssomyia intermedia | 75 |
| C4NAP5_DROME | Seminal fluid protein 93F | Drosophila melanogaster (Fruit fly) | 53 |
| E1JIS3_DROME | Seminal fluid protein 93F | Drosophila melanogaster (Fruit fly) | 53 |
| D9HQ83_HELME | Seminal fluid protein HACP033 | Heliconius melpomene (Postman butterfly) | 62 |
| SGP3_SCHGR | Serine protease inhibitor 3 (Protease inhibitor SGPI-3) (Serine protease inhibitor III) | Schistocerca gregaria (Desert locust) | 63 |
| A0A1W4WDB5_DROFC | serine protease inhibitor Kazal-type 1-like | Drosophila ficusphila (Fruit fly) | 62 |
| Q8T7L6_BOMMO | Silk proteinase inhibitor | Bombyx mori (Silk moth) | 65 |
| J9YLZ1_COPFO | Termicin | Coptotermes formosanus (Formosan subterranean termite) | 62 |
| A0A1C9UN27_CRYPU | Termicin | Cryptocercus punctulatus (Brown-hooded cockroach) | 61 |
| Q5SDH7_9NEOP | Termicin | Drepanotermes rubriceps | 62 |
| D2D021_9NEOP | Termicin | Macrotermes barneyi | 74 |
| D2D016_9NEOP | Termicin | Macrotermes barneyi | 62 |
| D2D009_9NEOP | Termicin | Macrotermes barneyi | 62 |
| D2D003_9NEOP | Termicin | Macrotermes barneyi | 62 |
| D2D018_9NEOP | Termicin | Macrotermes barneyi | 62 |
| D2D027_9NEOP | Termicin | Macrotermes barneyi | 62 |
| D2D053_9NEOP | Termicin | Macrotermes barneyi | 62 |
| D2D036_9NEOP | Termicin | Macrotermes barneyi | 62 |
| D2D044_9NEOP | Termicin | Macrotermes barneyi | 62 |
| D2D043_9NEOP | Termicin | Macrotermes barneyi | 62 |
| D2D015_9NEOP | Termicin | Macrotermes barneyi | 62 |
| D2D005_9NEOP | Termicin | Macrotermes barneyi | 62 |
| D2D050_9NEOP | Termicin | Macrotermes barneyi | 62 |
| D2D042_9NEOP | Termicin | Macrotermes barneyi | 62 |
| D2D028_9NEOP | Termicin | Macrotermes barneyi | 62 |
| D2D040_9NEOP | Termicin | Macrotermes barneyi | 62 |
| D2D031_9NEOP | Termicin | Macrotermes barneyi | 62 |
| D2D001_9NEOP | Termicin | Macrotermes barneyi | 62 |
| D2D029_9NEOP | Termicin | Macrotermes barneyi | 62 |
| D2D034_9NEOP | Termicin | Macrotermes barneyi | 62 |
| D2D010_9NEOP | Termicin | Macrotermes barneyi | 62 |
| D2D002_9NEOP | Termicin | Macrotermes barneyi | 62 |
| D2D030_9NEOP | Termicin | Macrotermes barneyi | 62 |
| D2D020_9NEOP | Termicin | Macrotermes barneyi | 62 |
| D2D033_9NEOP | Termicin | Macrotermes barneyi | 62 |
| D2D012_9NEOP | Termicin | Macrotermes barneyi | 74 |
| D2D004_9NEOP | Termicin | Macrotermes barneyi | 62 |
| D2D041_9NEOP | Termicin | Macrotermes barneyi | 62 |
| D2D024_9NEOP | Termicin | Macrotermes barneyi | 62 |
| D2D007_9NEOP | Termicin | Macrotermes barneyi | 62 |
| D2D039_9NEOP | Termicin | Macrotermes barneyi | 62 |
| D2D035_9NEOP | Termicin | Macrotermes barneyi | 69 |
| D2D032_9NEOP | Termicin | Macrotermes barneyi | 62 |
| D2D008_9NEOP | Termicin | Macrotermes barneyi | 62 |
| D2D026_9NEOP | Termicin | Macrotermes barneyi | 62 |
| D2D025_9NEOP | Termicin | Macrotermes barneyi | 62 |
| D2D023_9NEOP | Termicin | Macrotermes barneyi | 62 |
| D2D011_9NEOP | Termicin | Macrotermes barneyi | 62 |
| D2D014_9NEOP | Termicin | Macrotermes barneyi | 62 |
| D2D052_9NEOP | Termicin | Macrotermes barneyi | 62 |
| D2D022_9NEOP | Termicin | Macrotermes barneyi | 62 |
| D2D013_9NEOP | Termicin | Macrotermes barneyi | 62 |
| D2D017_9NEOP | Termicin | Macrotermes barneyi | 74 |
| Q5SDJ4_9NEOP | Termicin | Nasutitermes comatus | 62 |
| Q5SDJ5_9NEOP | Termicin | Nasutitermes comatus | 62 |
| Q5SDJ1_9NEOP | Termicin | Nasutitermes exitiosus | 62 |
| Q5SDI6_9NEOP | Termicin | Nasutitermes pluvialis | 62 |
| Q5SDI3_9NEOP | Termicin | Nasutitermes triodiae (Spinifex termite) | 59 |
| Q5SDI1_9NEOP | Termicin | Nasutitermes walkeri | 62 |
| Q5SDI0_9NEOP | Termicin | Nasutitermes walkeri | 62 |
| C9W4D2_9NEOP | Termicin | Odontotermes formosanus | 62 |
| C9W4D4_9NEOP | Termicin | Odontotermes formosanus | 62 |
| C9W4D5_9NEOP | Termicin | Odontotermes formosanus | 62 |
| C9W4D8_9NEOP | Termicin | Odontotermes formosanus | 62 |
| C9W4H5_9NEOP | Termicin | Odontotermes formosanus | 62 |
| C9W4E2_9NEOP | Termicin | Odontotermes formosanus | 62 |
| C9W4C3_9NEOP | Termicin | Odontotermes formosanus | 62 |
| C9W4F4_9NEOP | Termicin | Odontotermes formosanus | 62 |
| C9W4C9_9NEOP | Termicin | Odontotermes formosanus | 62 |
| C9W4G1_9NEOP | Termicin | Odontotermes formosanus | 62 |
| C9W4E9_9NEOP | Termicin | Odontotermes formosanus | 62 |
| C9W4E4_9NEOP | Termicin | Odontotermes formosanus | 62 |
| C9W4D7_9NEOP | Termicin | Odontotermes formosanus | 62 |
| C9W4C4_9NEOP | Termicin | Odontotermes formosanus | 62 |
| C9W4C5_9NEOP | Termicin | Odontotermes formosanus | 62 |
| C9W4E3_9NEOP | Termicin | Odontotermes formosanus | 62 |
| C9W4C6_9NEOP | Termicin | Odontotermes formosanus | 62 |
| C9W4F5_9NEOP | Termicin | Odontotermes formosanus | 74 |
| C9W4H7_9NEOP | Termicin | Odontotermes formosanus | 62 |
| C9W4C8_9NEOP | Termicin | Odontotermes formosanus | 62 |
| C9W4E1_9NEOP | Termicin | Odontotermes formosanus | 62 |
| C9W4G0_9NEOP | Termicin | Odontotermes formosanus | 62 |
| C9W4F1_9NEOP | Termicin | Odontotermes formosanus | 62 |
| C9W4G2_9NEOP | Termicin | Odontotermes formosanus | 62 |
| C9W4D0_9NEOP | Termicin | Odontotermes formosanus | 62 |
| C9W4D9_9NEOP | Termicin | Odontotermes formosanus | 62 |
| C9W4F2_9NEOP | Termicin | Odontotermes formosanus | 62 |
| C9W4F8_9NEOP | Termicin | Odontotermes formosanus | 62 |
| C9W4F6_9NEOP | Termicin | Odontotermes formosanus | 62 |
| C9W4E5_9NEOP | Termicin | Odontotermes formosanus | 62 |
| C9W4F7_9NEOP | Termicin | Odontotermes formosanus | 62 |
| C9W4E0_9NEOP | Termicin | Odontotermes formosanus | 62 |
| C9W4G3_9NEOP | Termicin | Odontotermes formosanus | 62 |
| C9W4G5_9NEOP | Termicin | Odontotermes formosanus | 62 |
| C9W4G8_9NEOP | Termicin | Odontotermes formosanus | 62 |
| C9W4D1_9NEOP | Termicin | Odontotermes formosanus | 62 |
| C9W4G9_9NEOP | Termicin | Odontotermes formosanus | 62 |
| C9W4G4_9NEOP | Termicin | Odontotermes formosanus | 62 |
| C9W4C7_9NEOP | Termicin | Odontotermes formosanus | 62 |
| C9W4G6_9NEOP | Termicin | Odontotermes formosanus | 62 |
| C9W4F3_9NEOP | Termicin | Odontotermes formosanus | 62 |
| C9W4E7_9NEOP | Termicin | Odontotermes formosanus | 62 |
| C9W4D3_9NEOP | Termicin | Odontotermes formosanus | 62 |
| C9W4E6_9NEOP | Termicin | Odontotermes formosanus | 62 |
| C9W4F0_9NEOP | Termicin | Odontotermes formosanus | 62 |
| C9W4F9_9NEOP | Termicin | Odontotermes formosanus | 74 |
| C9W4H0_9NEOP | Termicin | Odontotermes formosanus | 62 |
| C9W4D6_9NEOP | Termicin | Odontotermes formosanus | 62 |
| C9W4E8_9NEOP | Termicin | Odontotermes formosanus | 62 |
| C9W4J3_9NEOP | Termicin | Reticulitermes chinensis | 61 |
| C9W4I6_9NEOP | Termicin | Reticulitermes chinensis | 61 |
| C9W4J9_9NEOP | Termicin | Reticulitermes chinensis | 62 |
| C9W4K1_9NEOP | Termicin | Reticulitermes chinensis | 61 |
| C9W4J7_9NEOP | Termicin | Reticulitermes chinensis | 61 |
| C9W4I7_9NEOP | Termicin | Reticulitermes chinensis | 61 |
| C9W4J2_9NEOP | Termicin | Reticulitermes chinensis | 61 |
| C9W4J0_9NEOP | Termicin | Reticulitermes chinensis | 61 |
| C9W4J6_9NEOP | Termicin | Reticulitermes chinensis | 61 |
| C9W4L6_9NEOP | Termicin | Reticulitermes chinensis | 61 |
| C9W4K6_9NEOP | Termicin | Reticulitermes chinensis | 61 |
| C9W4K0_9NEOP | Termicin | Reticulitermes chinensis | 61 |
| C9W4K5_9NEOP | Termicin | Reticulitermes chinensis | 61 |
| D9D769_RETFL | Termicin | Reticulitermes flavipes (Eastern subterranean termite) | 61 |
| D9D771_RETFL | Termicin | Reticulitermes flavipes (Eastern subterranean termite) | 61 |
| D9D788_RETVI | Termicin | Reticulitermes virginicus (Dark southern subterranean termite) | 61 |
| D9D784_RETVI | Termicin | Reticulitermes virginicus (Dark southern subterranean termite) | 61 |
| D9D791_RETVI | Termicin | Reticulitermes virginicus (Dark southern subterranean termite) | 61 |
| Q5SDH8_9NEOP | Termicin | Tumulitermes pastinator | 62 |
| A0A1I8QAG7_STOCA | Uncharacterized protein | Stomoxys calcitrans (Stable fly) (Conops calcitrans) | 72 |
| A0A195ECR9_9HYME | Uncharacterized protein | Trachymyrmex cornetzi | 75 |
| W5JVP1_ANODA | Uncharacterized protein | Anopheles darlingi (Mosquito) | 75 |
| A0A0B4K6L9_DROME | Uncharacterized protein | Drosophila melanogaster (Fruit fly) | 53 |
| A0A0B4K6C0_DROME | Uncharacterized protein | Drosophila melanogaster (Fruit fly) | 56 |
| A0A0B4KEX7_DROME | Uncharacterized protein | Drosophila melanogaster (Fruit fly) | 62 |
| U4TW12_DENPD | Uncharacterized protein | Dendroctonus ponderosae (Mountain pine beetle) | 54 |
| U4TY91_DENPD | Uncharacterized protein | Dendroctonus ponderosae (Mountain pine beetle) | 61 |
| B3M6X8_DROAN | Uncharacterized protein | Drosophila ananassae (Fruit fly) | 69 |
| B3MVF1_DROAN | Uncharacterized protein | Drosophila ananassae (Fruit fly) | 75 |
| B3M6X7_DROAN | Uncharacterized protein | Drosophila ananassae (Fruit fly) | 69 |
| A0A0P9C2V6_DROAN | Uncharacterized protein | Drosophila ananassae (Fruit fly) | 75 |
| A0A0P8XLZ0_DROAN | Uncharacterized protein | Drosophila ananassae (Fruit fly) | 65 |
| B3NBV7_DROER | Uncharacterized protein | Drosophila erecta (Fruit fly) | 72 |
| B3NBV6_DROER | Uncharacterized protein | Drosophila erecta (Fruit fly) | 69 |
| B3NBU5_DROER | Uncharacterized protein | Drosophila erecta (Fruit fly) | 70 |
| B3NBU6_DROER | Uncharacterized protein | Drosophila erecta (Fruit fly) | 71 |
| B3NBU7_DROER | Uncharacterized protein | Drosophila erecta (Fruit fly) | 71 |
| B3NBU8_DROER | Uncharacterized protein | Drosophila erecta (Fruit fly) | 69 |
| B3NBV9_DROER | Uncharacterized protein | Drosophila erecta (Fruit fly) | 70 |
| A0A0Q5VLT1_DROER | Uncharacterized protein | Drosophila erecta (Fruit fly) | 62 |
| A0A0Q5VNI6_DROER | Uncharacterized protein | Drosophila erecta (Fruit fly) | 64 |
| A0A0Q9XLI4_DROMO | Uncharacterized protein | Drosophila mojavensis (Fruit fly) | 71 |
| A0A0Q9XH89_DROMO | Uncharacterized protein | Drosophila mojavensis (Fruit fly) | 68 |
| A0A0Q9X6V4_DROMO | Uncharacterized protein | Drosophila mojavensis (Fruit fly) | 62 |
| Q29LL5_DROPS | Uncharacterized protein | Drosophila pseudoobscura pseudoobscura (Fruit fly) | 74 |
| B5DYJ6_DROPS | Uncharacterized protein | Drosophila pseudoobscura pseudoobscura (Fruit fly) | 71 |
| A0A0R3NR47_DROPS | Uncharacterized protein | Drosophila pseudoobscura pseudoobscura (Fruit fly) | 65 |
| A0A0J9TLN1_DROSI | Uncharacterized protein | Drosophila simulans (Fruit fly) | 66 |
| A0A0J9QW55_DROSI | Uncharacterized protein | Drosophila simulans (Fruit fly) | 73 |
| A0A0J9RER0_DROSI | Uncharacterized protein | Drosophila simulans (Fruit fly) | 62 |
| B4NKR2_DROWI | Uncharacterized protein | Drosophila willistoni (Fruit fly) | 64 |
| B4P1K4_DROYA | Uncharacterized protein | Drosophila yakuba (Fruit fly) | 68 |
| B4PF50_DROYA | Uncharacterized protein | Drosophila yakuba (Fruit fly) | 74 |
| B4PGP8_DROYA | Uncharacterized protein | Drosophila yakuba (Fruit fly) | 72 |
| B4PGP7_DROYA | Uncharacterized protein | Drosophila yakuba (Fruit fly) | 70 |
| B4PGN7_DROYA | Uncharacterized protein | Drosophila yakuba (Fruit fly) | 70 |
| B4PGN8_DROYA | Uncharacterized protein | Drosophila yakuba (Fruit fly) | 71 |
| B4PGN9_DROYA | Uncharacterized protein | Drosophila yakuba (Fruit fly) | 69 |
| B4PGQ0_DROYA | Uncharacterized protein | Drosophila yakuba (Fruit fly) | 70 |
| B4PF53_DROYA | Uncharacterized protein | Drosophila yakuba (Fruit fly) | 74 |
| A0A0R1E0M7_DROYA | Uncharacterized protein | Drosophila yakuba (Fruit fly) | 69 |
| A0A0R1EA48_DROYA | Uncharacterized protein | Drosophila yakuba (Fruit fly) | 66 |
| A0A0R1DSV7_DROYA | Uncharacterized protein | Drosophila yakuba (Fruit fly) | 64 |
| A0A0R1E1L3_DROYA | Uncharacterized protein | Drosophila yakuba (Fruit fly) | 53 |
| E2AKX3_CAMFO | Uncharacterized protein | Camponotus floridanus (Florida carpenter ant) | 47 |
| E2B985_HARSA | Uncharacterized protein | Harpegnathos saltator (Jerdon's jumping ant) | 68 |
| E2B8E0_HARSA | Uncharacterized protein | Harpegnathos saltator (Jerdon's jumping ant) | 68 |
| G6CML0_DANPL | Uncharacterized protein | Danaus plexippus (Monarch butterfly) | 65 |
| A0A067R348_ZOONE | Uncharacterized protein | Zootermopsis nevadensis (Dampwood termite) | 69 |
| A0A067QRV6_ZOONE | Uncharacterized protein | Zootermopsis nevadensis (Dampwood termite) | 71 |
| A0A067QM12_ZOONE | Uncharacterized protein | Zootermopsis nevadensis (Dampwood termite) | 75 |
| M9NEQ6_DROME | Uncharacterized protein | Drosophila melanogaster (Fruit fly) | 73 |
| K7INT1_NASVI | Uncharacterized protein | Nasonia vitripennis (Parasitic wasp) | 70 |
| K7J442_NASVI | Uncharacterized protein | Nasonia vitripennis (Parasitic wasp) | 60 |
| K7IVK7_NASVI | Uncharacterized protein | Nasonia vitripennis (Parasitic wasp) | 66 |
| K7J9G8_NASVI | Uncharacterized protein | Nasonia vitripennis (Parasitic wasp) | 75 |
| K7IRW4_NASVI | Uncharacterized protein | Nasonia vitripennis (Parasitic wasp) | 63 |
| J9L5P0_ACYPI | Uncharacterized protein | Acyrthosiphon pisum (Pea aphid) | 68 |
| K7JDC5_NASVI | Uncharacterized protein | Nasonia vitripennis (Parasitic wasp) | 69 |
| K7JTI8_NASVI | Uncharacterized protein | Nasonia vitripennis (Parasitic wasp) | 69 |
| A0A194QPM1_PAPXU | Uncharacterized protein | Papilio xuthus (Asian swallowtail butterfly) | 73 |
| A0A0L7QXG3_9HYME | Uncharacterized protein | Habropoda laboriosa | 51 |
| A0A0L7QTM6_9HYME | Uncharacterized protein | Habropoda laboriosa | 75 |
| A0A154PK86_9HYME | Uncharacterized protein | Dufourea novaeangliae | 54 |
| A0A154P498_9HYME | Uncharacterized protein | Dufourea novaeangliae | 56 |
| A0A026WD17_CERBI | Uncharacterized protein | Cerapachys biroi (Ant) | 73 |
| A0A026WW26_CERBI | Uncharacterized protein | Cerapachys biroi (Ant) | 65 |
| J9KHE3_ACYPI | Uncharacterized protein | Acyrthosiphon pisum (Pea aphid) | 63 |
| X1XDA5_ACYPI | Uncharacterized protein | Acyrthosiphon pisum (Pea aphid) | 62 |
| A0A182GT03_AEDAL | Uncharacterized protein | Aedes albopictus (Asian tiger mosquito) (Stegomyia albopicta) | 75 |
| A0A182FGK9_ANOAL | Uncharacterized protein | Anopheles albimanus (New world malaria mosquito) | 65 |
| A0A182IFU6_ANOAR | Uncharacterized protein | Anopheles arabiensis (Mosquito) | 57 |
| A0A182I4E8_ANOAR | Uncharacterized protein | Anopheles arabiensis (Mosquito) | 70 |
| A0A182LD39_9DIPT | Uncharacterized protein | Anopheles coluzzii | 70 |
| A0A182NDL0_9DIPT | Uncharacterized protein | Anopheles dirus | 66 |
| A0A182S0S6_ANOFN | Uncharacterized protein | Anopheles funestus (African malaria mosquito) | 68 |
| A0A182S2D7_ANOFN | Uncharacterized protein | Anopheles funestus (African malaria mosquito) | 47 |
| A0A182RZB0_ANOFN | Uncharacterized protein | Anopheles funestus (African malaria mosquito) | 63 |
| A0A1I8JV96_ANOME | Uncharacterized protein | Anopheles merus (Mosquito) | 68 |
| A0A182VXB3_9DIPT | Uncharacterized protein | Anopheles minimus | 64 |
| A0A182WQ44_9DIPT | Uncharacterized protein | Anopheles minimus | 44 |
| A0A182X4E8_ANOQN | Uncharacterized protein | Anopheles quadriannulatus (Mosquito) | 70 |
| A0A182XS18_ANOQN | Uncharacterized protein | Anopheles quadriannulatus (Mosquito) | 47 |
| A0A182XSA8_ANOQN | Uncharacterized protein | Anopheles quadriannulatus (Mosquito) | 68 |
| A0A182YJ77_ANOST | Uncharacterized protein | Anopheles stephensi (Indo-Pakistan malaria mosquito) | 65 |
| A0A088ATK2_APIME | Uncharacterized protein | Apis mellifera (Honeybee) | 75 |
| A0A1B0BNW8_9MUSC | Uncharacterized protein | Glossina palpalis gambiensis | 59 |
| A0A1B0C0M4_9MUSC | Uncharacterized protein | Glossina palpalis gambiensis | 73 |
| Q2QKD5_MAYDE | Uncharacterized protein | Mayetiola destructor (Hessian fly) | 62 |
| T1GEA2_MEGSC | Uncharacterized protein | Megaselia scalaris (Humpbacked fly) (Phora scalaris) | 57 |
| D5LXI2_9HYME | Uncharacterized protein | Nylanderia nr. pubens LZ-2010 | 68 |
| F1CZR3_9HYME | Uncharacterized protein | Nylanderia nr. pubens LZ-2010 | 70 |
| I4DLP0_PAPXU | Uncharacterized protein | Papilio xuthus (Asian swallowtail butterfly) | 53 |
| A0A1W4U5N7_DROFC | uncharacterized protein LOC108086447 | Drosophila ficusphila (Fruit fly) | 64 |
| A0A0B4K755_DROME | Uncharacterized protein, isoform A | Drosophila melanogaster (Fruit fly) | 64 |
| A0A0B4LET5_DROME | Uncharacterized protein, isoform B | Drosophila melanogaster (Fruit fly) | 62 |
| A0A151X356_9HYME | Venom protease inhibitor 2 | Trachymyrmex zeteki | 59 |
| A0A034WXR3_APHER | Venom toxin-like peptide | Aphidius ervi (Aphid parasite) | 60 |
| A0A034WY34_APHER | Venom toxin-like peptide | Aphidius ervi (Aphid parasite) | 61 |
| A0A034WWW1_APHER | Venom toxin-like peptide | Aphidius ervi (Aphid parasite) | 51 |

**Table S2.** Top 5 templates selected by Swiss-Model for constructing the structural model of nine mature iTOLIP mini-protein.

| >sp\|H9KQJ7\|21-74 | 54AA |  |
| --- | --- | --- |
| ASKCGRHGDSCVSSSDCCPGTWCHTYANRCQVRITEEELMKQREKILGRKGKDY | | |
| Template | **Sequence Identity** | **Description** |
| 2n86.1.A | 44.12 | Spiderine-1a |
| 2jtk.1.A | 38.24 | Dickkopf-related protein 2 |
| 1lmr.1.A | 42.42 | TOXIN ADO1 |
| 1nix.1.A | 34.38 | HAINANTOXIN-I |
| 1qdp.1.A | 38.71 | ROBUSTOXIN |
| >tr\|A0A084WJA1\|26-71 | 47AA |  |
| DSICENGSTPEAAELCKARCDTLKFRYSFCFNGDCKCAGPIVGRTD | | |
| Template | **Sequence Identity** | **Description** |
| 2b68.1.A | 24.14 | defensin |
| 2e3e.1.A | 31.03 | defensin, mutant DEF-BBB |
| 2e3g.1.A | 27.59 | defensin, mutant DEF-DAA |
| 2ln4.1.A | 27.59 | Coprisin |
| 2e3f.1.A | 28.57 | defensin, mutant DEF-BAT |
| >tr\|J7HBU2\|24-70 | 47AA |  |
| EDQSLDRSKRACRYFFGNCQHDPCCEHLFCNSLKFCGWDAAYTIRKG | | |
| Template | **Sequence Identity** | **Description** |
| 5t4r.1.A | 51.52 | Mu-theraphotoxin-Pn3a |
| 2n6o.1.A | 48.48 | Kappa-theraphotoxin-Hm1a |
| 1la4.1.A | 50 | SGTx1 |
| 1d1h.1.A | 45.45 | HANATOXIN TYPE 1 |
| 2a2v.1.A | 50 | Jingzhaotoxin-XI |
| >tr\|J7HIK0\|24-70 | 47AA |  |
| EDQSLDRSKRACRYFFGNCQHDPCCEHLFCNSLKFCGWDAAYTIRKG | | |
| Template | **Sequence Identity** | **Description** |
| 5t4r.1.A | 51.52 | Mu-theraphotoxin-Pn3a |
| 2n6o.1.A | 48.48 | Kappa-theraphotoxin-Hm1a |
| 1la4.1.A | 50 | SGTx1 |
| 1d1h.1.A | 45.45 | HANATOXIN TYPE 1 |
| 2a2v.1.A | 50 | Jingzhaotoxin-XI |
| J7HBS6\|21-70 | 50AA |  |
| SPYNLPEDHSLSRAKRDTCRYLFGWCGNGEKCCPHLGCGGMLFCVWDGKV | | |
| Template | **Sequence Identity** | **Description** |
| 5t4r.1.A | 51.52 | Mu-theraphotoxin-Pn3a |
| 1la4.1.A | 51.52 | SGTx1 |
| 2n6o.1.A | 48.48 | Kappa-theraphotoxin-Hm1a |
| 2a2v.1.A | 45.45 | Jingzhaotoxin-XI |
| 2m9l.1.A | 48.48 | Beta-theraphotoxin-Tp1a |
| >tr\|J7HBT1\|26-75 | 50AA |  |
| FQEDHTLVLAETPKKACRWFWGTCSKTEDCCKHLSCNFLGLCDLTVAKRN | | |
| Template | **Sequence Identity** | **Description** |
| 1d1h.1.A | 46.67 | HANATOXIN TYPE 1 |
| 1la4.1.A | 46.67 | SGTx1 |
| 2n6o.1.A | 50 | Kappa-theraphotoxin-Hm1a |
| 5t4r.1.A | 46.67 | Mu-theraphotoxin-Pn3a |
| 1koz.1.A | 48.28 | Voltage-dependent Channel Inhibitor |
| >tr\|A0A034WXR3\|25-60 | 36AA |  |
| DAVCKRPGEICDPKEELNKCCIGKCQAIGGNPGKCI | | |
| Template | **Sequence Identity** | **Description** |
| 1q3j.1.A | 33.33 | ALO3 |
| 1tt3.1.A | 44 | Omega-conotoxin MVIIa |
| 1dw4.1.A | 44 | OMEGA-CONOTOXIN MVIIA |
| 1dw5.1.A | 44 | OMEGA-CONOTOXIN MVIIA |
| 1ttk.1.A | 44 | Omega-conotoxin MVIIa |
| >tr\|A0A034WY34\|25-61 | 37AA |  |
| YKGCRRLGSKCHPTLKPCCSALTCKPIDGDNGECVEK | | |
| Template | **Sequence Identity** | **Description** |
| 2lqa.1.A | 43.75 | Asteropsin A |
| 2lzx.1.A | 43.75 | Asteropsin B |
| 3q8j.1.A | 43.75 | Asteropsin A |
| 2e2s.1.A | 33.33 | Agelenin |
| 2h1z.1.A | 31.25 | Hybrid atracotoxin |
| >tr\|A0A034WWW1\|25-51 | 26AA |  |
| YCLGSGKQCGKFDDCCSLTCMSNGECA | |  |
| Template | **Sequence Identity** | **Description** |
| 1omn.1.A | 48 | OMEGA-CONOTOXIN M VII C (M SEVEN C) |
| 1cnn.1.A | 48 | OMEGA-CONOTOXIN MVIIC |
| 1mvj.1.A | 44 | SVIB |
| 1v4q.1.A | 44 | omega-conotoxin MVIIC |
| 1lmr.1.A | 42.31 | TOXIN ADO1 |

**Figure S1. Scoring of ClanTox predictions for insects’ secreted mini-proteins.** Distribution of ClanTox predictions of 4180 insects’ secreted proteins shorter than 75 AA. The top scoring iTOLIPs are marked by P3 (dark red), the intermediate confidence is P2 and P1 is the least confident predictions. The gray marks the bulk of the sequences (76%) with negative prediction (i.e. not a toxin- like protein). All together there are 379 proteins that are scored as P3 (Table S1).
